# Dissection of Neurochemical Pathways Across Complexity and Scale

**DOI:** 10.1111/jnc.70160

**Published:** 2025-07-21

**Authors:** Alice Abbondanza, Nawon Kim, Ricardo A. S. Lima‐Filho, Azin Amin, Roberta G. Anversa, Felipe Borges Almeida, Pablo L. Cardozo, Giovanna Carello‐Collar, Emma V. Carsana, Royhaan O. Folarin, Sara Guerreiro, Olayemi K. Ijomone, Sodiq K. Lawal, Isadora Matias, Smart I. Mbagwu, Sandra A. Niño, Bolanle F. Olabiyi, Sunday Y. Olatunji, Tosin A. Olasehinde, Waralee Ruankham, William N. Sanchez, Carina Soares‐Cunha, Paula A. Soto, Jazmín Soto‐Verdugo, Nathan R. Strogulski, Weronika Tomaszewska, Cármen Vieira, Adriano Chaves‐Filho, Michael A. Cousin, Ago Rinken, Tyler J. Wenzel

**Affiliations:** ^1^ Laboratory of Neurochemistry Institute of Physiology of the Czech Academy of Sciences Prague Czech Republic; ^2^ CNRS, Inserm, Institut de Biologie Paris Seine (IBPS), Centre de Neuroscience Sorbonne Université (NeuroSU), Sorbonne Université Paris France; ^3^ Centre for Discovery Brain Sciences University of Edinburgh Edinburgh UK; ^4^ Muir Maxwell Epilepsy Centre University of Edinburgh Edinburgh UK; ^5^ Simons Initiative for the Developing Brain University of Edinburgh Edinburgh UK; ^6^ Institute of Medical Biochemistry Leopoldo De Meis Federal University of Rio de Janeiro Rio de Janeiro Brazil; ^7^ Florey Institute of Neuroscience and Mental Health, University of Melbourne Melbourne Victoria Australia; ^8^ Graduate Program in Health Sciences Federal University of Health Sciences of Porto Alegre (UFCSPA) Porto Alegre Brazil; ^9^ Department of Biochemistry and Immunology Institute of Biological Sciences, Universidade Federal de Minas Gerais Belo Horizonte Brazil; ^10^ Graduate Program in Biological Sciences: Biochemistry, Department of Biochemistry Institute of Health Basic Sciences, Universidade Federal do Rio Grande do Sul (UFRGS) Porto Alegre Brazil; ^11^ Department of Medical Biotechnology and Translational Medicine University of Milan Milan Italy; ^12^ Division of BioMedical Sciences University of Global Health Equity Butaro Rwanda; ^13^ Life and Health Sciences Research Institute (ICVS), School of Medicine University of Minho Braga Portugal; ^14^ ICVS/3B's—PT Government Associate Laboratory Braga Portugal; ^15^ Food Chemistry, Faculty of Mathematics and Natural Science University of Wuppertal Wuppertal Germany; ^16^ Department of Anatomy University of Medical Sciences Ondo Nigeria; ^17^ School of Nursing Faculty of Health Sciences, University of Botswana Garborone Botswana; ^18^ Institute of Biomedical Sciences Federal University of Rio de Janeiro Rio de Janeiro Brazil; ^19^ Department of Anatomy, Faculty of Basic Medical Sciences Nnamdi Azikiwe University Anambra Nigeria; ^20^ Geroscience Center for Brain Health and Metabolism (GERO) Santiago Chile; ^21^ Department of Biology, Faculty of Sciences Universidad de Chile Santiago Chile; ^22^ Medical Faculty, Institute of Molecular Psychiatry University of Bonn Bonn Germany; ^23^ The Babraham Institute Signalling Program Cambridge UK; ^24^ Department of Pharmacology Vanderbilt University Brain Institute, Vanderbilt University Nashville Tennessee USA; ^25^ Nutrition and Toxicology Division, Food Technology Department Federal Institute of Industrial Research Lagos Nigeria; ^26^ Discipline of Microbiology School of Life Sciences, University of Kwazulu‐Natal Durban South Africa; ^27^ Department of Clinical Chemistry, Faculty of Medical Technology Mahidol University Bangkok Thailand; ^28^ Integrative Neurobiology Section National Institute on Drug Abuse Intramural Research Program Baltimore Maryland USA; ^29^ Universidad de Buenos Aires. CONICET. Instituto de Química y Fisicoquímica Biológicas (IQUIFIB) Buenos Aires Argentina; ^30^ Department of Anatomy and Neurobiology Virginia Commonwealth University School of Medicine Richmond Virginia USA; ^31^ Neurotrauma and Neuroimmunology Research Group Trinity College Dublin, University of Dublin Dublin Ireland; ^32^ Laboratory for Translational Research in Neuropsychiatric Disorders (TREND) Nencki Institute of Experimental Biology Warsaw Poland; ^33^ Translational Neuropsychiatry Group Łukasiewicz Research Network ‐ PORT Polish Center for Technology Development Wroclaw Poland; ^34^ University of Victoria Victoria British Columbia Canada; ^35^ Institute of Chemistry, University of Tartu Tartu Estonia; ^36^ Department of Psychiatry College of Medicine, University of Saskatchewan Saskatoon Saskatchewan Canada

**Keywords:** biosensors, *C. elegans*, *Drosophila*, gene therapy, iPSCs, super‐resolution microscopy

## Abstract

The field of Neurochemistry spent decades trying to understand how the brain works, from nano to macroscale and across diverse species. Technological advancements over the years allowed researchers to better visualize and understand the cellular processes underpinning central nervous system (CNS) function. This review provides an overview of how novel models, and tools have allowed Neurochemistry researchers to investigate new and exciting research questions. We discuss the merits and demerits of different in vivo models (e.g., *Caenorhabditis elegans* , *Drosophila melanogaster* , *Ratus norvegicus*, and *Mus musculus* ) as well as in vitro models (e.g., primary cells, induced pluripotent stem cells, and immortalized cells) to study Neurochemical events. We also discuss how these models can be paired with cutting‐edge genetic manipulation (e.g., CRISPR‐Cas9 and engineered viral vectors) and imaging techniques, such as super‐resolution microscopy and new biosensors, to study cellular processes of the CNS. These technological advancements provide new insight into Neurochemical events in physiological and pathological contexts, paving the way for the development of new treatments (e.g., cell and gene therapies or small molecules) that aim to treat neurological disorders by reverting the CNS to its homeostatic state.

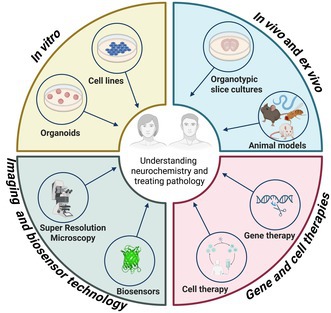

## Graphical abstract

The field of Neurochemistry spent decades trying to understand how the brain works, and many recent technological advancements have allowed researchers to unlock some mysteries of the brain. This review, which was written by attendees of the Advanced School hosted by the International Society of Neurochemistry in 2023, summarizes these technological advancements as well as their advantages and disadvantages. The tools described in this review provide new insight into health and disease and also pave the way for the development of new treatments that aim to treat brain diseases by having the brain return to its normal, healthy state.

**Abbreviations**
BRET: bioluminescence resonance energy transfer
cAMP: cyclic adenosine monophosphate
CNS: central nervous system
CRISPR: clustered regularly interspaced short palindromic repeats
DREADDs: designer receptors exclusively activated by designer drugs
EM: electron microscopy
FRET: Förster resonance energy transfer
GABA: γ‐aminobutyric acid
GPCR: G‐protein‐coupled receptors
iPSC: induced pluripotent stem cells
PA‐GFP: photoactivable green fluorescent protein
PAINT: point accumulation for imaging in nanoscale topography
PALM: photoactivated localization microscopy
RESOLFT: reversible saturable optically linear fluorescence transitions microscopy
rsFP: reversibly switchable fluorescent proteins
SRM: super‐resolution microscopy
STED: stimulated emission depletion microscopy
STORM: stochastic optical reconstruction microscopy
TR‐FRET: time‐resolved FRET

## 1 Introduction

The human nervous system consists of billions of neurons and glia, and the journey to understand how they interact with each other has been ongoing for more than a century. When this complexity is coupled with the ethical issues prohibiting human experiments, it is obvious that alternative approaches are needed. This review discusses the different in vivo models, such as *Caenorhabditis elegans* (roundworm), *Drosophila melanogaster* (fruit fly, hereafter referred to as *Drosophila*), *Ratus norvegicus* (rat), *and Mus musculus* (mouse), as well as in vitro models, such as primary cells, immortalized cells, and induced pluripotent stem cells (iPSCs), that have been used to better understand Neurochemistry. We also discuss cutting‐edge tools, such as super‐resolution imaging and new probes, that have given the field of Neurochemistry new insights into the inner workings of the nervous system. The advantages and disadvantages of these models and tools are discussed, and we provide key examples demonstrating their uses for investigating the processes involved in Neurochemical events. In brief, these models and new advancements in technology have given researchers unprecedented spatial and temporal resolution to visualize these events in real time. We end this review by discussing how the new insights offered by these tools and models will allow the field to develop better treatments for diseases.

This review was written by the attendees of the Advanced School hosted by the International Society of Neurochemistry in 2023. In this Advanced School, attendees learned about new tools and techniques that have facilitated new discoveries in the field of Neurochemistry. This review is the culmination of topics taught and discussed at the School on “New challenges and opportunities in Neurochemical studies—novel tools and approaches,” and the aim was to provide a review and critical appraisal of these topics tailored toward scientists entering the field of Neurochemistry.

## 2 In Vitro Models

In vitro cultures are the most convenient way to observe and manipulate neurochemical events due to the ease of data acquisition and analysis in these models. Many different culture systems exist; however, we will discuss the four most commonly used in vitro models, namely primary cell cultures, immortalized cell line cultures, organotypic slice cultures, and iPSCs. For each model, we will provide specific examples to explain their advantages and limitations as models for studying Neurochemistry, and for convenience, we have summarized this information in Table 1.

**TABLE 1:** Summary of the advantages and limitations of different in vitro models.

	Advantages	Limitations
Primary cells	No exogenous factors needed to differentiate cells Can be derived from animals of different age Little influence by genetic drift and contamination that may arise at high passage numbers Same genetic background as donor	May need exogenous factors in media to maintain cell phenotype Limited or no proliferation Variability between preparations Phenotype in culture deviates from in situ state
Immortalized cells	Low cost Near‐indefinite proliferation High transfection efficiency due to short doubling times Homogenous culture with similar genetic background Highly reproducible responses due to culture homogeneity	Phenotype influenced by cancer immortalization or viral genetic reprogramming May need exogenous factors to differentiate cells into a more relevant phenotype Highly susceptible to genetic drift and cell contamination that may arise at high passage numbers
Organotypic brain slice culture	Contains physiological circuits and brain structure Provides a brain microenvironment most similar to the in vivo brain	Lack of published experiments to validate the model as translatable to physiological and pathological contexts Cell death and circuit disruption resulting from axotomy Requires highly optimized culture media Unclear how long‐term maintenance affects culture characteristics
Induced pluripotent stem cells (iPSCs)	Similar genetic background as the human or mouse donor Can produce relatively homogenous monocultures of any cell type Can be manipulated into brain organoids to generate a culture with a three‐dimensional brain‐like microenvironment Retains genetic heterogeneity of donors, and thus can be used to test whether results are generalizable across genetic backgrounds Differentiation can be shortened using gene transduction methods Can be used to replace dysfunctional cell types in the brain to fix issues arising from genetic mutations	Generally considered high‐cost cultures, especially if studies aim to capture donor heterogeneity or use exogenous factors to differentiate cultures Cells differentiated in different batches have been considered to be heterogeneous Many weeks to generate desired cultures if using exogenous factors Unknown consequence of gene transduction methods of cell phenotype

### 2.1 Primary Cell Cultures

Primary cells refer to cells isolated from a tissue, and existing protocols can isolate and culture almost any given central nervous system (CNS) cell, such as neurons (Sahu et al. 2019), microglia (Bohlen et al. 2019), astrocytes (Schildge et al. 2013), and oligodendrocyte precursor cells (OPCs) (Chen et al. 2007). There are several techniques to isolate primary cells from the tissue, including using density gradient centrifugation (Chen et al. 2007), immunopanning (Zhang et al. 2016), fluorescence‐activated or magnetic‐activated cell sorting (Bohlen et al. 2019; Kim et al. 2012), or cell‐specific cytotoxic drugs (Sahu et al. 2019). An advantage of primary cell cultures is that they can be isolated from human or animal tissue of different ages (Gradisnik et al. 2020; Park et al. 2020; Vijaya et al. 2023), allowing the study of aging‐related questions (Gerrits et al. 2020; Crain et al. 2013; Park et al. 2020). In contrast, one limitation for primary cell cultures is the difficulty in obtaining pure cultures. Primary cultures are often mixed with multiple cell types, which can make it difficult to attribute data to a specific cell type. For example, cultures with 95% astrocytes and 5% microglia gave results that were different from those with higher purity (Baxter et al. 2021). However, this limitation can also be an advantage since the results of these experiments may be more relevant to an in vivo study where cell–cell crosstalk occurs. Another challenge of the primary culture is their limited proliferation, which requires multiple preparations and donor organisms to obtain the requisite cells to address specific research questions. For example, adult neurons, microglia, and astrocytes often have limited or no capacity to proliferate (Stansley et al. 2012; Belmonte‐Mateos and Pujades 2022; Ciccarelli et al. 2000). Although the need to cultivate new primary cultures overcomes a limitation of immortalized cell lines by preventing genetic drift and reducing the risk of cell line contamination, which ultimately safeguards data integrity. Ultimately, primary cell cultures approximate the characteristics of their in situ counterparts and can account for age as a biological variable in studies (Galatro et al. 2017; Wenzel et al. 2023)—with the primary drawback being the extra labor and resources required to generate these cultures.

### 2.2 Immortalized Cell Lines

Immortalized cell lines, such as human SH‐SY5Y neuroblastomas and U138 MG glioblastomas, are derived from tumors while others, such as the HMC3 microglia and the SVG p12 astrocyte lines, are immortalized by the simian virus 40 (Carter et al. 2022; Dello Russo et al. 2018; Kulshreshtha and Agrawal 2020). Immortalized cells maintain features similar to their primary cell counterparts, but with the added convenience of being able to proliferate indefinitely (Kovalevich and Langford 2013; Galland et al. 2019; Cheepsunthorn et al. 2001; Louis et al. 1992). The high proliferation rate of immortalized cells makes them particularly suited to be used in transfection experiments, as researchers can quickly establish how genes affect neurochemical events (Jordan et al. 2008). However, it is often unclear how the immortalization process affects cell physiology (Wenzel et al. 2023), and thus data derived from these cultures may have limited relevance to the physiological context. For example, PC12 cells are pheochromocytoma cells derived from an adrenal gland tumor and have a phenotype that is a mix of multiple cells. To illustrate this, PC12 cells differentiated into a neuronal phenotype in the presence of nerve growth factor show cotransmission of dopamine, norepinephrine, and acetylcholine, which is a phenomenon not reported in primary neurons (Vaaga et al. 2014; El Mestikawy et al. 2011; Svensson et al. 2019). As another example, immortalized astrocyte and glioma cell lines have reduced glutamate metabolism compared to primary astrocytes (Galland et al. 2019). Another limitation of cell immortalization is that the resulting cultures are more susceptible to genetic drift and cell contamination (Allen et al. 2016; Soice and Johnston 2021). Despite their limitations, immortalized cell lines have many experimental uses, such as their aforementioned benefit for studies using transfections; although, researchers need to be diligent to ensure best cell culture practices are followed, such as recording passage number, obtaining cell lines from a reliable source, and confirming genomic integrity (Pamies 2021).

### 2.3 Organotypic Brain Slice Culture

Organotypic brain slice cultures are 100–400 μm thick tissue slices of living animal or human brain tissue that can be maintained ex vivo for varying periods of time (Eugène et al. 2014; Humpel 2015; Schwarz et al. 2017; Verwer et al. 2003; Lyman et al. 1991). These cultures are particularly advantageous because they maintain neural circuitry, which is a feature lost in dissociated culture (Stoppini et al. 1991; Duff et al. 2002; Bahr 1995; Gähwiler 1981). Because these features make organotypic slice cultures similar to an actual brain environment, they have been useful for studies investigating neural networks and circuits (Manz et al. 2021; Qi et al. 2015) as well as brain metabolism (Das et al. 2020; Andersen et al. 2021; Sapir et al. 2021). Moreover, exogenous factors can be applied to organotypic slice cultures as easily as cell lines, making them much easier to work with than mammalian animal models. Despite their advantages, there are many aspects of organotypic slice cultures that remain uncertain, such as the development of these tissues when cultured ex vivo. For example, to our best knowledge, no study has characterized the maturation of brain slice cultures derived from neonatal or early‐postnatal tissues in an ex vivo environment (Humpel 2015), and thus researchers must still validate that their findings in vivo (Croft et al. 2019; Humpel 2015). Another challenge is the excessive cell death in slice cultures derived from adult brain samples (Humpel 2015), and this challenge may exist because the media for organotypic slice cultures requires optimization, as many formulations induce necrosis with prolonged culture (van der Valk et al. 2010; Chen et al. 2008). There are also inherent limitations to organotypic slice models, such as the cell death resulting from axotomy, or the loss of efferent or afferent circuits previously established in the organ (Humpel 2015). In summary, due to technical difficulties, slice cultures are not widely used even though they allow access to circuits.

### 2.4 Induced Pluripotent Stem Cells

IPSCs are somatic cells that have been reprogrammed to become pluripotent. They are commonly of human origin, although mouse iPSCs are also used. IPSCs have been successfully differentiated into many different types of brain cells, including neurons (Schwartzentruber et al. 2018; Bianchi et al. 2018; Sasaki et al. 2019), as well as the major glial cell types astrocytes, microglia, and oligodendrocytes (summarized in Wenzel et al. 2023). These cells can either be used to generate relatively pure two‐dimensional cultures of brain cell types, or they can be manipulated to differentiate into a three‐dimensional brain‐like tissue called brain organoids (different protocols to generate brain organoids are reviewed elsewhere Wenzel and Mousseau 2025). Interestingly, brain organoids can be generated from human or mouse iPSCs using highly similar protocols (Sánchez et al. 2024), indicating similar signals govern the brain development of these species. Largely, the physiology of brain organoids and organotypic slice cultures is comparable (Sánchez et al. 2024), as is protein processing in both brain organoids and brain (Wenzel and Mousseau 2025). Both the three‐dimensional and two‐dimensional cultures derived from iPSCs are useful for modeling disease and physiology, screening drugs, and studying brain development (van der Kant et al. 2019; Birnbaum et al. 2018; Arber et al. 2021; Wang, Ward, et al. 2017; Oakley et al. 2021; Chadarevian et al. 2022). A major downside of iPSC cultures is that they often take many weeks to mature into the required cell type or a mature three‐dimensional tissue, with costly reagents present in the culture medium. However, many research groups are developing methods that induce transgene expression to greatly shorten the time for these cultures to reach maturity, reducing their cost (Lendemeijer et al. 2024; Cakir et al. 2022; Zhang et al. 2013; Ehrlich et al. 2017). Similar to primary cell cultures, a limitation of iPSC cultures is their genetic heterogeneity if multiple cell lines are being used, but this can be accounted for in a robust experimental design (reviewed in Wenzel and Mousseau 2025). Power analyses have also been completed for different experimental designs, including isogenic and case–control designs (Brunner et al. 2023). Overall, iPSC cultures can model many different aspects of the brain in a physiological and pathological context, such as cell–cell signaling, brain development and metabolism, and are a versatile tool for Neurochemistry studies.

## 3 In Vivo Models

A variety of in vivo models exist, and they each come with varying degrees of complexity for the study of neurochemical events (Manger et al. 2008; Żakowski 2020). For example, *Drosophila* can be considered a less complex animal when compared to primates or humans due to their small genome as well as their simplified nervous system and social behavior. For similar reasons, *C. elegans* are also widely used in neurochemical research. For example, *C. elegans* were used to validate that a mutation in the *MYCBP2* gene was the cause of some humans exhibiting highly superior autobiographical memory (Papassotiropoulos et al. 2024). Nonetheless, rodent models, such as the rat and mice, are the most commonly used in vivo models in Neurochemistry studies because they have many similarities with higher order animals, such as humans. Here, we provide specific examples demonstrating the uses of *C. elegans* , *Drosophila*, mice, and rats to answer Neurochemistry questions, and summarize the advantages and disadvantages as well as the characteristics of these models in Table 2.

**TABLE 2:** Summary of the advantages and limitations of different in vivo models.

	Advantages	Limitations
*C. elegans* 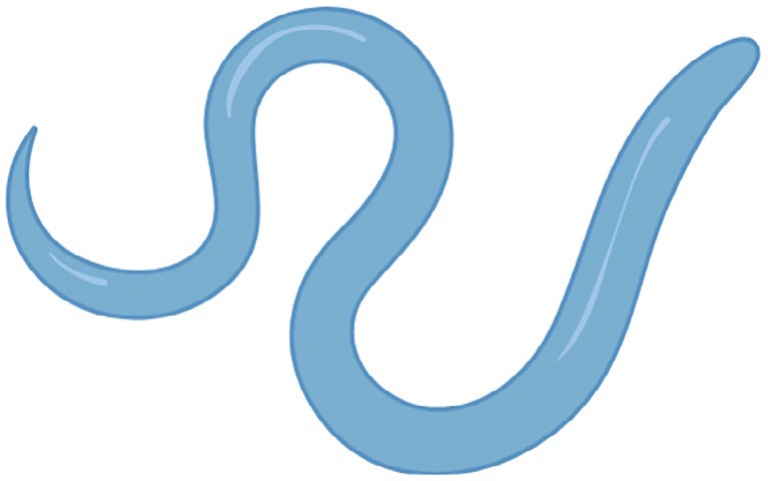	Can create genetic models quickly due to short lifecycle High numbers of offspring make it easier to power experiments Excellent for genetic screening studies Transparent body allows easy access to cellular and subcellular structural visualization	Small genome with limited orthologs to other species Simple nervous system limits research questions that can be answered Fundamentals of neural activity are different from mammals Lack of blood–brain barrier‐like structure Lack of myelinated neurons
*Drosophila* 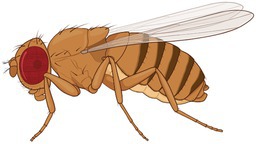	Can create genetic models quickly due to short lifecycle High numbers of offspring make it easier to power experiments Has most neurotransmitters found in mammals Has a blood–brain barrier‐like structure Excellent for genetic screening studies	Nervous system is still simplified compared with higher order animals Fundamentals of neural activity are different from mammals Lack of myelinated neurons
Mice and rats 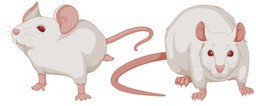	Highly complex nervous system and social behaviors that justifies their use in a wide variety of experiments Has a blood–brain barrier More sophisticated gene editing systems and probes available for the rodent nervous system Similarities with higher order animals: Presence of essential cell types Similar principles of neural activity Similar proteins and signaling mechanisms Many genes are orthologous	Costly Highly complex nervous system and social behaviors can complicate study design and data acquisition Limited offspring mean it takes over a year to generate and validate new genetic models Gene editing systems are more complex to use
Similarities across these models	Fully mapped genome and connectome Models share similar cellular processes Models can be used in behavioral studies	Brain structure, cell composition and pathways are not identical to humans Results may have limited translation to the human context

### 3.1 *C. elegans*

*C. elegans* are a low‐cost in vivo model with a simplified nervous system containing 302 to 385 neurons (depending on its life stage), making it popular for studies investigating the function of genes (Kim et al. 2018). Illustrating their use for studying neurochemical functions, *C. elegans* have sensory, motor, and interneurons that form chemical synapses, neuromuscular junctions, and gap junctions. Importantly, the connections that these neurons make have been fully mapped (Cook et al. 2019). It is important to note that *C. elegans* do not have myelinated neurons, and their neurons fire continuously, which is different from mammalian neurons, which fire in a binary manner (Oikonomou and Shaham 2011; Lindsay et al. 2011). They have also been used to study synaptic vesicle recycling (Mizumoto et al. 2023), and there are many biological tags or reporter molecules that can be used to image neuronal activity (Clark et al. 2023; Ashley et al. 2021; Wang, Tang, et al. 2017; Wester et al. 2023). In fact, *C. elegans* were pivotal in understanding the molecules involved in axonal migration (Branda and Stern 1999; Teixeira‐Castro et al. 2023). It is also possible to image the entire neuronal activity of *C. elegans* (Atanas et al. 2023) and, when coupled with tools like NeuroPAL, which creates a multicolor fluorescent map of neurons (Yemini et al. 2021), researchers can visualize the neuronal activation patterns in response to a variety of factors, such as heat, odors, feeding, and changes in locomotion. Furthermore, *C. elegans* share many cellular processes that more complex animals have, such as processes related to cell division, apoptosis, aging, and cell signaling; however, only around 30 of the 534 *C. elegans* genes are homologous with those of more complex animals like humans (Kim et al. 2018; Boulin and Hobert 2012), potentially limiting the translation of certain genetic studies. Yet, the short life cycle of *C. elegans* also provides an advantage for screening gene function. Thus, they have been used in many experiments using RNA interference and clustered regularly interspaced short palindromic repeats‐Cas9 (CRISPR‐Cas9) systems (Kutscher and Shaham 2014; Nance and Frøkjær‐Jensen 2019).

*C. elegans* can also be used for behavioral studies as well as drug screening. Indeed, *C. elegans* models of Alzheimer disease, which carry a mutation in the gene orthologous to human amyloid precursor protein and apolipoprotein E, can be used to model a decline in locomotion (Yi et al. 2017; Griffin et al. 2019). Similar behavioral studies with *C. elegans* have been used to study Huntington's disease (Machiela et al. 2021), and chemotaxic studies have been employed to study memory and learning (Papassotiropoulos et al. 2024; Gourgou et al. 2021). When behavioral studies are coupled with exposure to exogenous compounds, *C. elegans* can also be used to test the efficacy and neurotoxicity of the exposure (Simonetta and Golombek 2007; Stroustrup et al. 2013; Youssef et al. 2019; Mondal et al. 2016). Furthermore, due to their short life cycle, they can be exploited for testing drugs at a higher‐throughput than traditional animal models, such as mice and rats (Petrascheck et al. 2007; Gosai et al. 2010; Teixeira‐Castro et al. 2023; Sohrabi et al. 2021). For example, *C. elegans* played a pivotal role in identifying how the serotonin transporter is targeted by selective serotonin reuptake inhibitors (Ranganathan et al. 2001).

### 3.2 Drosophila

*Drosophila* are in vivo models that are often associated with improving our understanding of genes and genetic heritability (Adams et al. 2000), but there has been a renewed interest in this organism for neurochemical studies due to their short lifespan, orthologous genes with humans, and functional nervous system (Adams et al. 2000; Pandey and Nichols 2011; Hanesch et al. 1989; Sugie et al. 2018). Similar to *C. elegans, Drosophila* neurons fire passively and continuously (Akin et al. 2019) and lack myelination. However, a potential advantage of *Drosophila* over *C. elegans* is their more complicated nervous system; they have over 100 000 neurons, and their CNS can be divided into four distinct regions (Hanesch et al. 1989; Sugie et al. 2018). *Drosophila* also have synaptic processes that are similar to more complex animals, such as glutamate, γ‐aminobutyric acid (GABA), and acetylcholine signaling (Sugie et al. 2018). Demonstrating the use of these signaling systems in *Drosophila*, the activity of three specific GABAergic and cholinergic neurons has been shown to control walking, halting, and grooming (Sapkal et al. 2024). Taken together, *Drosophila* are a widely used and cost‐effective model for understanding the effects of different molecules on neuronal activity or synaptic activity.

*Drosophila* have also been used for behavioral studies, with these studies able to link neurochemical events with behavioral responses. For example, *Drosophila* were employed to investigate behaviors such as courtship, locomotion, learning, memory, and social interactions (Mollá‐Albaladejo and Sánchez‐Alcañiz 2021; Rooke et al. 2020; Bentzur et al. 2021). Researchers have been able to induce learned helplessness and aggressive behavior in *Drosophila* by exposing them to stressors (Yang et al. 2013; Rasti et al. 2020; Kravitz and Fernandez 2015), demonstrating their use in determining how exogenous exposures can affect complex behavioral responses. Similarly, *Drosophila* behavior can be reinforced through environmental stressors such as heat (Sitaraman et al. 2008), indicating that they can be used as a model to study the relationship between rewards and neurotransmitter release. *Drosophila* are also an excellent model system for forward genetic screening. For example, a series of key synaptic genes required for synaptic vesicle recycling and proteostasis were identified via mutagenic screens targeted to the eye of the organism (Uytterhoeven et al. 2011; Verstreken et al. 2009). This strategy circumvented the potential lethality of an organism‐wide approach. In summary, *Drosophila* can be used for many of the same studies as *C. elegans*, but their more complicated neural network and larger genome add complexity—approximating this model to higher order animals, such as rodents and humans.

### 3.3 Mice and Rats

Mice and rats are commonly used for Neurochemistry studies due to their structural and cellular similarities with humans (Hodge et al. 2019; Kim et al. 2023; Beauchamp et al. 2022; Xu et al. 2022). However, there are structural differences between rodents and larger mammals such as primates (Smart et al. 2002), as well as differences in cell composition and the number of synapses (Loomba et al. 2022). It is also important to note that while mice and rats are often grouped together under the term rodents, there are differences between them. For example, rats have more accelerated neurogenesis in the hippocampus compared to mice and exhibit more social behaviors, which may make them a more relevant model for studying social disorders (Snyder et al. 2009). Nonetheless, rats and mice can be used to observe the same neurochemical events, with the same techniques used to manipulate and image them. In fact, many of the same techniques used in *C. elegans* and *Drosophila* can be used in mice and rats.

Gene editing systems can be used with rodents to better understand the link between a gene and a neurochemical system, and the systems available for rodents are often very sophisticated. For example, a technique called designer receptors exclusively activated by designer drugs (DREADDs) generates synthetic receptors in rodents that can be used to precisely manipulate different circuits (Armbruster et al. 2007; Vlasov et al. 2018). The principle of the DREADD technique is that the synthetic receptors have no endogenous ligand, and thus can only be activated upon the delivery of a synthetic ligand. Several neurochemical studies have taken advantage of this powerful technique. These studies uncovered the roles of neuronal circuits and glia in the manifestation of complex behaviors in mice, such as the expression of fear in a fear conditioning paradigm (Martin‐Fernandez et al. 2017). The DREADD technique also uncovered the circuits involved in compulsive behavior (King et al. 2021), stress‐induced binge eating (G. Anversa et al. 2023), pain sensitization (Patra et al. 2023), and epileptiform activity (Wenker et al. 2022).

While rodents are a highly valuable model for neurochemical studies, their translational value recently came into question, as several drug candidates and therapeutic approaches that have shown promising results in mice failed to demonstrate safety or efficacy in human clinical trials for CNS disorders (Franco and Cedazo‐Minguez 2014; Zahs and Ashe 2010; Azkona and Sanchez‐Pernaute 2022). These failures raised questions regarding the fidelity of mouse models to accurately model human conditions, including neurological and neuropsychiatric disorders (Białoń and Wąsik 2022). Multiple factors may contribute to these incongruities of data from different species, such as interspecies differences in transcriptional and epigenetic differences, regional brain volume, and the circuitry in brain regions responsible for higher cognitive functions such as the prefrontal cortex (Lin et al. 2014; Wong et al. 2023; DeFelipe 2011; Rakic 2009; Molnár and Clowry 2012). To overcome the potential lack of translation of rodent data to the human context, novel approaches, such as the application of Research Domain Criteria for animal studies, have been proposed to unequivocally improve the translatability of results to the human context (for a comprehensive review see Anderzhanova et al. 2017).

## 4 Imaging

Historically, neurochemical processes could only be interrogated via classical protein biochemistry techniques, limiting the ability to visualize these events in both time and space. The advent of fluorescence imaging revealed the intricate interplay of neurochemical signaling and events in ever‐increasing spatiotemporal resolution. Some of the key technologies that precipitated these advances are outlined below.

### 4.1 Introduction to Super‐Resolution Imaging

Super‐resolution imaging was developed to overcome the Abbe limit, which is on the level of 200 nm maximum resolution of conventional light microscopy, and to visualize subcellular structures. Electron microscopy (EM) can obtain a resolution of 2 nm, which allowed researchers to image nanoscale components, such as intracellular organelles and membrane conformations (Kater et al. 2023; Penczek 2010; Bernard et al. 1997). However, EM has limitations, such as a need for absolute dehydration of any prepared specimen (Tuijtel et al. 2019). Super‐resolution microscopy (SRM) has not quite reached the resolution of EM, but certain SRM tools have come extraordinarily close with a resolution of 10 nm (Arizono et al. 2023; Fuhrmann et al. 2022).

The resolution offered by SRM provides great insight into neurochemical processes. In addition to being able to image subcellular structures, SRM allows neurochemists to track cellular dynamics of brain processes (Arizono et al. 2023; Fuhrmann et al. 2022). The advantages and disadvantages of some of the most popular SRM techniques are summarized in Table 3, and we discuss examples that demonstrate how different types of SRM are used to study Neurochemistry below.

**TABLE 3:** Summary of the advantages and limitations of different super‐resolution imaging techniques, as well as their underlying principles.

Method	Principle	Advantages	Limitations
STED 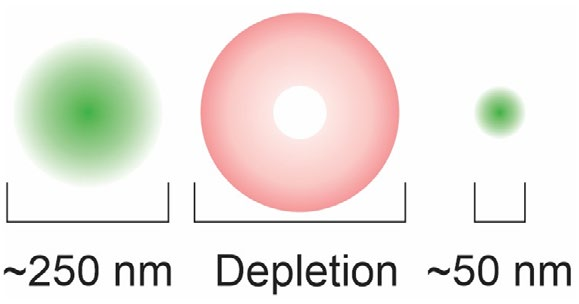	Builds upon the principles of fluorescence and stimulated emission. The premise is to use stimulated emission to “deplete” fluorescence emission from areas beyond the central focal point, resulting in a smaller effective point spread function and better spatial resolution	Allows live cell imaging Live 3D imaging High spatial resolution Confocal speed acquisition No data processing	High photobleaching Phototoxicity Limited axial resolution Photostable labelling required
RESOLFT 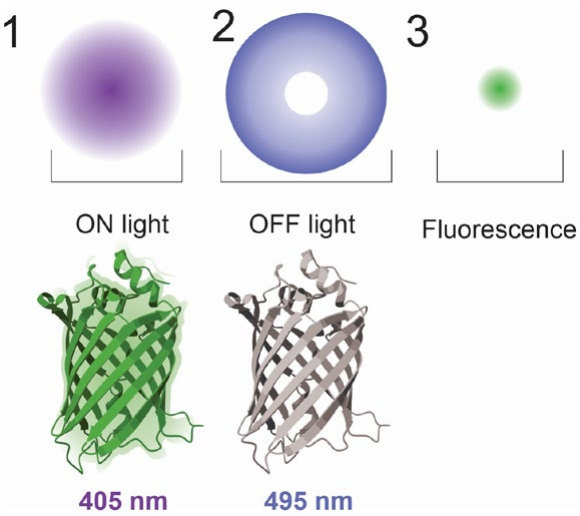	Involves switching the fluorophores between two states: a fluorescent and a nonfluorescent. By accurately controlling the transition between these states, precise manipulation of the fluorescence can be achieved, resulting in highly detailed images	Allows live cell imaging Live 3D imaging High‐speed imaging No data processing	Limited axial resolution Reversible switchable proteins required for labelling
STORM 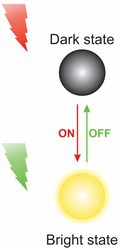	Employs pairs of fluorophores, to induce photoswitching of the reporter molecule when thiols are present in an oxygen‐scavenging buffer. This process transitions the reporter from a bright “active” state to a dimmer, longer lived “inactive” state. Simultaneously, the activator molecule is used to efficiently reactivate the reporter fluorophore.	High localization precision Live 3D imaging Easy sample preparation Favorable signal‐to‐noise ratio	Limited live cell imaging High labelling density Long acquisition times Computational artifacts
PALM 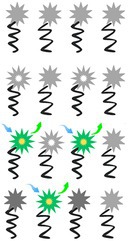	Relies on the photoactivation or photoconversion of fluorescent proteins to create a limited and sparse subset of fluorescent molecules in each frame of the imaging process.	Allows live cell imaging Live 3D imaging Quantification Protein imaging	Low localization precision High labelling density Long acquisition times Computational artifacts Complex data processing
PAINT 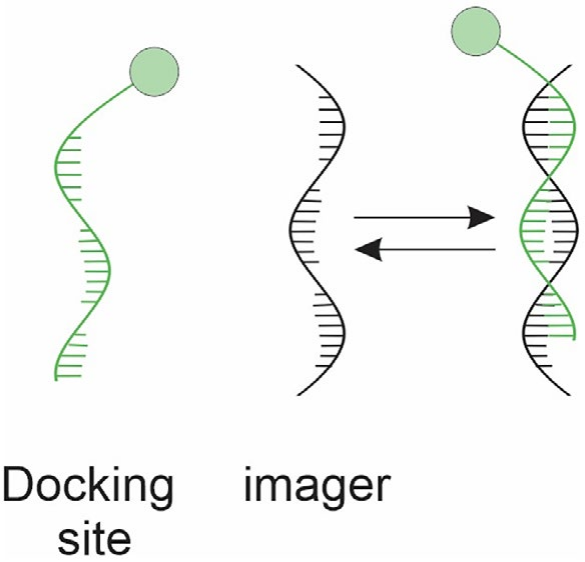	Uses individual fluorophores to reversibly bind to the target structure, which are switched to their “active” state and detected, alternating between two states: free diffusion and immobilization through binding to a specific target	High localization precision No photobleaching Endogenous labelling Allows multiplexing	Very limited live cell imaging Background noise System stability Long acquisition times Complex data processing

Abbreviations: PAINT, point accumulation for imaging in nanoscale topography; PALM, photoactivated localization microscopy; RESOLFT, reversible saturable optically linear fluorescence transitions microscopy; STED, stimulated emission depletion microscopy; STORM, stochastic optical reconstruction microscopy.

### 4.2 Deterministic Methods Such as STED and RESOLFT

Deterministic super‐resolution techniques, such as *stimulated emission depletion (STED) microscopy* or *reversible saturable/switchable linear optical fluorescence transition (RESOLFT)*, consist of methods developed to selectively restrict the excitation of a limited region of fluorophores in a specimen (Hell and Wichmann 1994; Godin et al. 2014). Controlling the excitation patterns to circumscribe the fluorescent region minimizes the cross‐sectional area of the focal point to a size below the diffraction barrier, and thus enhances the achievable resolution to as low as 10 nm (Cristofari et al. 2022; Westphal et al. 2008; Nägerl et al. 2008; Bancelin et al. 2023).

STED microscopy consists of a laser scanning confocal method where a second doughnut‐shaped depletion beam is overlapped, at a different wavelength, on top of the excitation laser beam. This depletes the excitation of the fluorophore to a focal spot that is less than 50 nm. The ability to resolve structures less than 50 nm led to the use of STED to study the dynamics of synapses and synaptic vesicles, as well as the dynamics of ion channels and chromatin (Kostiuk et al. 2019; Cristofari et al. 2022; Rutherford 2015). While STED microscopy is user‐friendly, relatively fast, and effective at optical sectioning, it suffers from phototoxicity due to the amount of laser power needed (Arizono et al. 2023).

RESOLFT was a method aimed to mitigate phototoxicity arising from STED microscopy by using special fluorophores called reversibly switchable fluorescent proteins (rsFPs), thus reducing laser power (Godin et al. 2014). These proteins are special variations of broadly used fluorescent proteins, such as Dronpa, EGFP, YFP, or mCherry, combined with a photoreceptor derived by the photoreceptor protein YtvA from *Bacillus subtilis* (Fuhrmann et al. 2022; Kwon et al. 2015; Hell 2009). While RESOLFT was successful in reducing phototoxicity, there are fewer fluorescent proteins commercially available for RESOLFT than fluorophores available for STED microscopy (Kwon et al. 2015). In part, this is due to the rapid photobleaching of fluorophores that are not rsFPs when imaged using RESOLFT techniques.

### 4.3 Stochastic Methods Such as STORM, PALM, and PAINT

Methods, such as stochastic optical reconstruction microscopy (STORM), photoactivated localization microscopy (PALM), point accumulation for imaging in nanoscale topography (PAINT), involve: (1) stochastic activation and identification of individual fluorescent molecules across multiple frames to reconstruct high‐resolution images; and (2) the use of advanced analysis algorithms to extract quantitative information across the multiple frames (Lelek et al. 2021). Stochastic methods deliberately activate a limited number of fluorophores in each camera frame, allowing the precise position of fluorophores, and then the final image is reconstructed by integrating the multiple frames (Hell 2009). The reconstructed image can have a resolution as low as 10 nm (Liu et al. 2022).

STORM employs a pair of fluorophores (i.e., an activator and a reporter) that can change their photochemical properties (e.g., fluorescence, charge, and hydrophilicity) in the presence of redox agents such as catalase or glucose oxidase (Dempsey et al. 2009; Huang et al. 2008; Bates et al. 2007). The resolution limit of STORM is approximately 20–30 nm. Similar to other SRM methods, STORM proved useful for imaging the organization of proteins at synaptic terminals, including receptors, transporters, and vesicular cargo (Igarashi et al. 2018; Carvalhais et al. 2021; Pennacchietti et al. 2017; Jones et al. 2017; Badawi and Nishimune 2020).

PALM relies on the photoactivation or photoconversion of genetically modified fluorescent proteins to create a limited and sparse subset of fluorescent molecules in each frame of the imaging process (Hess et al. 2006; Betzig et al. 2006; Hess et al. 2007). The resolution of PALM is between 10 to 30 nm (Lippincott‐Schwartz and Patterson 2009). Fluorescent proteins may be photoactivatable fluorescent proteins, such as PA‐GFP, or photoswitchable fluorescent proteins, such as Dronpa and rsCherry (Flors et al. 2007; Patterson and Lippincott‐Schwartz 2002). These fluorescent proteins typically exist in an “inactive” state, characterized by low fluorescence or darkness, and can be induced to become fluorescent or shift their emission wavelength when exposed to an activation light.

PAINT employs individual fluorophores, which can reversibly bind to the target structure. Upon binding with the target, they acquire an “active” conformation and can be detected. Unbound fluorophores remain in the “inactive” state and therefore are not detected (Sharonov and Hochstrasser 2006; Lelek et al. 2021). DNA‐PAINT, the main variant of this method, attaches a short single‐stranded DNA molecule, known as the docking strand, to the target protein or structure of interest. A complementary DNA strand labeled with a fluorescent dye, referred to as imager strands, diffuses freely within the sample and binds to the docking strand. Most importantly, DNA‐PAINT is suitable for multiplexing, offering a theoretically unlimited capacity for analyzing a variety of probes simultaneously (Jungmann et al. 2014).

The field of SRM is rapidly advancing, with new methods and tools being integrated into Neurochemistry studies frequently. Before planning a study, however, it is crucial to thoroughly understand the capabilities and limitations of each method (Yang et al. 2021; Jacquemet et al. 2020; Arizono et al. 2023). Specifically, it is important to remember that the “eternal triangle” of fluorescent microscopy—balancing sensitivity, resolution, and speed—also applies to SRM, necessitating careful compromises (Schermelleh et al. 2019).

### 4.4 Other Modern Tools to Visualize Neurochemical Events

To understand signal transduction mechanisms and pathways in the brain, several tools have been developed, which can be used with a conventional spectrofluorometer, fluorescent microscope, and SRM.

A wide range of fluorescent protein‐based sensors have been designed to monitor changes in the concentration and localization of various ions. In addition to Ca^2+^ sensors—which have been available since the late 20th century—constructs now exist for detecting K^+^, Mg^2+^, Cu^2+^, Cl,^−^ Zn^2+^, and other ions (Aper et al. 2016; Baek et al. 2021; Miyawaki et al. 1997). The autofluorescence from endogenous cellular proteins limits the use of these sensors more widely in tissues, but this limitation can be minimized by employing red‐shifted fluorescent sensors (Fink et al. 2025).

In addition to some ions, cyclic adenosine monophosphate (cAMP) also plays a crucial role as a secondary messenger in cellular signaling. To date, more than 80 genetically encoded biosensors have been developed to monitor cAMP dynamics both in vitro and in vivo (Massengill et al. 2021). These biosensors are primarily based on cAMP‐binding proteins such as Epac or protein kinase A, fused with fluorescent proteins that form Förster resonance energy transfer (FRET) pairs. While many of these sensors perform well in vitro, achieving a sufficient signal‐to‐noise ratio under physiological conditions in vivo remains a significant challenge.

A novel step in the visualization of neurotransmitter molecules was achieved with the implementation of G‐protein‐coupled receptor (GPCR)‐based genetically encoded fluorescent indicators (Rohner et al. 2024). Here, specific ligand binding by the receptor is converted into a change in the fluorescent signal, which allows direct detection of the neurotransmitter. The list of target molecules includes dopamine, serotonin, acetylcholine, norepinephrine, and histamine, and this list continues to grow.

Immunohistochemistry has been the gold standard for decades for neurochemical studies, including the detection of components of signal transduction pathways in the brain (Hara et al. 2024; Kalyuzhny 2011). It has been complemented by radioligand binding to cells and autoradiography on tissue sections. In recent decades, a long list of fluorescent‐labeled high‐affinity specific ligands for various receptors has been developed (Szabó et al. 2025).

Flow cytometry (Cunningham 2010), fluorescence polarization and anisotropy (Rinken et al. 2018), as well as direct determination of the intensity of fluorescent ligands on the cell surface (Allikalt et al. 2021), have become more widely used to characterize ligand binding affinity and kinetics to their receptors. To increase the sensitivity and throughput of the assay, several genetically modified constructs of receptors have been developed. These include FRET and time‐resolved FRET (TR‐FRET), where a second fluorophore or lanthanide cryptate is coupled with the receptor (Albizu et al. 2010). An advanced approach is the nano‐bioluminescence resonance energy transfer (nanoBRET) technique, where a bioluminescent protein (NanoLuc) is expressed on the N‐terminus of the target protein (Stoddart et al. 2018).

Another series of genetically encoded reporters has revealed key insights into the neurochemical mechanisms that occur at the synapse. One key advance was the visualization of the trafficking of synaptic vesicle cargo proteins during exocytosis and endocytosis. This was achieved using genetically encoded reporters called pHluorins, which consist of an exogenously expressed synaptic vesicle protein with an exquisitely pH‐sensitive EGFP (pHluorin) fused to a lumenal domain (Miesenböck et al. 1998). The acidic interior of the synaptic vesicle (required to drive uptake of neurotransmitter) quenches the pHluorin moiety reporter; however, during neurotransmitter release, it encounters the neutral synaptic cleft, and its dequenching reports vesicle fusion. Since their advent over 25 years ago, pHluorins have been critical in reporting essential molecular mechanisms that underpin both synaptic vesicle exocytosis and endocytosis (reviewed in Harper and Smillie 2021; Kavalali and Jorgensen 2014).

## 5 Future Directions: Leveraging Neurochemical Insights to Treat Diseases

New tools, such as imaging technologies and model systems, have clearly provided new insights into neurochemical events in both physiological and pathological contexts. These insights will hopefully provide the impetus for the discovery of treatments for a variety of neurological disorders. By identifying neurochemical abnormalities in a pathological CNS, researchers are aiming to correct neurological disorders by reverting the CNS to its original homeostatic state. Classically, small molecules have been used to treat neurological disorders, but recently, cell and gene therapies are gaining prominence as effective treatments for many of these disorders (Björklund and Lindvall 2017; Cartier et al. 2009; Konstantinidis et al. 2022).

CeIl and gene therapies are two types of treatments that fall under the broad umbrella of regenerative medicine. Broadly, cell therapies involve the addition of healthy cells or replacement of dysfunctional ones in the CNS to fix cellular mechanisms that become dysregulated by disease processes. Recently, these healthy cells have been sourced by differentiating iPSCs into the cell type of interest, rather than using a primary tissue source. Gene therapies deliver genetic material into human cells that replace a disease‐causing genetic mutation with a gene without adverse mutations. These therapies, their delivery methods, and the diseases they can cure have been exhaustively reviewed elsewhere (Hoang et al. 2022; Bulcha et al. 2021). Table 4 provides a brief overview of the advantages and disadvantages of the different gene therapy tools, while Table 5 summarizes the source tissues commonly used for cell therapies.

**TABLE 4:** Summary of the advantages and limitations of different primary gene therapy tools.

Direct editing tools	Advantages	Limitations
Gene delivered by nonintegrating (episomal) plasmids	Affordable costs Low pathogenicity, immunological effect, and oncogenic potential Can deliver a variety of transgene sizes	Limited ability to edit genes
Gene delivered by lentivirus	Can deliver transgenes up to 13 kb, but generally up to 8 kb is used	Pathogenic potential makes production more complicated Capacity to self‐replicate
Gene delivered by adeno‐associated virus	Cannot self‐replicate	Transgenes limited to ~4.7 kb Pathogenic potential makes production more complicated, but less pathogenic to humans than lentiviruses
Gene delivered by adenoviruses	Can deliver transgenes up to ~40 kb	Pathogenic potential makes production more complicated
All gene editing tools	Long‐lasting gene expression Can edit genes of nondividing cells	Concerns regarding liver damage when used in gene therapies for human disease

**TABLE 5:** Summary of the advantages and limitations of different source tissues to develop cell therapies.

Cell therapies	Advantages	Limitations
Induced pluripotent stem cells (iPSCs)	Can differentiate into any cell type, enabling the replacement of specific cell niches No ethical concerns regarding their origin Cells can be edited with direct editing tools for the creation of autologous cell therapies Cells can be derived from healthy donors and used as an allogeneic cell therapy Hypoallogeneic iPSC lines exist to limit immune responses in recipients Indefinite ability to expand in vitro	Require more sophisticated logistics and costly quality control to ensure no contamination Potential for recipient of cells to exhibit an immune response High passage numbers of pluripotent cells in vitro may result in chromosomal aberrations
Embryonic stem cells	Pluripotent and can differentiate into any cell type, enabling the replacement of specific cell niches Indefinite ability to expand in vitro	Ethical concerns regarding their origin Due to their origin, any potential cell therapies would be allogeneic in nature Potential for recipient of cells to exhibit an immune response High passage numbers of pluripotent cells in vitro may result in chromosomal aberrations
Mesenchymal stem cells	Cells can be edited with direct editing tools for the creation of autologous cell therapies Cells can be derived from healthy donors and used as an allogeneic cell therapy	Highly heterogenous cell type, with broad defining characteristics Multipotent and therefore unable to differentiate into all cell types Potential for recipient of cells to exhibit an immune response, although regarded as safer Limited ability to expand in vitro

There are many examples demonstrating how Neurochemical discoveries are leading to cell and gene therapy treatments for diseases. For instance, the Neurochemical basis of Parkinson's disease, namely the dopaminergic deficiencies, was discovered seven decades ago in the 1950s (Goetz 2011); shortly thereafter in the early 1960s, L‐DOPA was first used in humans to treat the symptoms of Parkinson's disease (Birkmayer and Hornykiewicz 1961) and research began to maximize the efficacy of this treatment (Pfeiffer and Ebadi 1972). Since the late 1970s, cell therapies aimed to repopulate the dopaminergic cell niche and restore dopamine synthesis in the brain started being developed (Björklund and Lindvall 2017). In 1999, the first human patient received a dopaminergic cell therapy and went into remission for many years, although low doses of L‐DOPA were introduced after 6 years (Björklund and Lindvall 2017). As another example still needing further investigation, preclinical in vitro studies introducing microglia that are homozygous for the nonpathogenic apolipoprotein E ε3 allele into tissues carrying the apolipoprotein E ε4 (a risk allele for Alzheimer's disease) report that the tissues display less β‐amyloid_1‐42_ accumulation (Lin et al. 2018). Gene therapies have also shown promise. For example, they have been used to treat the neurometabolic disorder X‐linked adrenoleukodystrophy, as demonstrated by patients displaying similar outcomes in a 2‐year follow‐up to those who receiving the traditional hematopoietic stem cell transplant treatment (Cartier et al. 2009). Preclinical in vitro studies have also suggested that patients with genetic forms of Alzheimer's disease may be protected by gene editing. For example, fibroblasts with the presenilin 1 M146L mutation—a mutation responsible for early‐onset Alzheimer's disease—produce more β‐amyloid_1‐42_ than the same fibroblasts that had the presenilin 1 gene corrected to the wildtype allele by a CRISPR‐Cas9 gene editing system (Konstantinidis et al. 2022). While gene therapies are proving to be a powerful tool for protecting patients against disease symptoms, adverse events, such as liver damage, are an ongoing concern (Jagadisan and Dhawan 2023; Kaiser et al. 2019). Furthermore, different vectors of gene therapies have limitations to the size of genes that can be delivered, and thus not every disease arising from a mutation may be able to be treated with this methodological approach (Counsell et al. 2017). For many different diseases, it is worth testing whether cell therapies can offer therapeutic benefits without the need for gene editing.

New tools to study Neurochemistry have unlocked the ability to answer exciting research questions, bringing unique insights into the pathogenesis of different diseases. With a renewed understanding of cellular dysfunction in disease, which may be the result of genetic mutations, cell senescence, or exposures, Neurochemists may be able to turn their attention to treating diseases with cell and gene therapies. Due to the increasing regulatory approval of using cell and gene therapies in humans, we have never been closer to the widespread use of these unique therapies (Bulaklak and Gersbach 2020). It is no longer a question of if these therapies will treat human disease, but when these therapies will be approved to treat human disease (Elverum and Whitman 2020).

## Author Contributions

**Alice Abbondanza:** writing – original draft, writing – review and editing. **Nawon Kim:** writing – original draft, writing – review and editing. **Ricardo A. S. Lima‐Filho:** writing – original draft, writing – review and editing. **Azin Amin:** writing – original draft. **Roberta G. Anversa:** writing – original draft. **Felipe Borges Almeida:** writing – original draft. **Pablo L. Cardozo:** writing – original draft. **Giovanna Carello‐Collar:** writing – original draft. **Emma V. Carsana:** writing – original draft. **Royhaan O. Folarin:** writing – original draft. **Sara Guerreiro:** writing – original draft. **Olayemi K. Ijomone:** writing – original draft. **Sodiq K. Lawal:** writing – original draft. **Isadora Matias:** writing – original draft. **Smart I. Mbagwu:** writing – original draft. **Sandra A. Niño:** writing – original draft. **Bolanle F. Olabiyi:** writing – original draft. **Sunday Y. Olatunji:** writing – original draft. **Tosin A. Olasehinde:** writing – original draft. **Waralee Ruankham:** writing – original draft. **William N. Sanchez:** writing – original draft. **Carina Soares‐Cunha:** writing – original draft. **Paula A. Soto:** writing – original draft. **Jazmín Soto‐Verdugo:** writing – original draft. **Nathan R. Strogulski:** writing – original draft. **Weronika Tomaszewska:** writing – original draft. **Cármen Vieira:** writing – original draft. **Adriano Chaves‐Filho:** writing – original draft. **Michael A. Cousin:** writing – original draft, writing – review and editing. **Ago Rinken:** writing – review and editing, writing – original draft. **Tyler J. Wenzel:** writing – original draft, writing – review and editing.

## Conflicts of Interest

Michael A. Cousin is a handling editor for Journal of Neurochemistry.

## Peer Review

The peer review history for this article is available at https://www.webofscience.com/api/gateway/wos/peer‐review/10.1111/jnc.70160.

## Data Availability

Data sharing not applicable to this article as no datasets were generated or analysed during the current study.

## References

[jnc70160-bib-0001] Adams, M. D. , S. E. Celniker , R. A. Holt , et al. 2000. “The Genome Sequence of *Drosophila melanogaster* .” Science 287: 2185–2195. 10731132 10.1126/science.287.5461.2185

[jnc70160-bib-0002] Akin, O. , B. T. Bajar , M. F. Keles , M. A. Frye , and S. L. Zipursky . 2019. “Cell‐Type Specific Patterned Stimulus‐Independent Neuronal Activity in the Drosophila Visual System During Synapse Formation.” Neuron 101: 894–904.e5. 30711355 10.1016/j.neuron.2019.01.008 PMC6437771

[jnc70160-bib-0003] Albizu, L. , M. Cottet , M. Kralikova , et al. 2010. “Time‐Resolved FRET Between GPCR Ligands Reveals Oligomers in Native Tissues.” Nature Chemical Biology 6: 587–594. 20622858 10.1038/nchembio.396 PMC3506176

[jnc70160-bib-0004] Allen, M. , M. Bjerke , H. Edlund , S. Nelander , and B. Westermark . 2016. “Origin of the U87MG Glioma Cell Line: Good News and Bad News.” Science Translational Medicine 8: 354re3. 10.1126/scitranslmed.aaf6853 27582061

[jnc70160-bib-0005] Allikalt, A. , T. Laasfeld , M. Ilisson , S. Kopanchuk , and A. Rinken . 2021. “Quantitative Analysis of Fluorescent Ligand Binding to Dopamine D3 Receptors Using Live‐Cell Microscopy.” FEBS Journal 288: 1514–1532. 32783364 10.1111/febs.15519

[jnc70160-bib-0006] Andersen, J. V. , E. W. Westi , E. Jakobsen , N. Urruticoechea , K. Borges , and B. I. Aldana . 2021. “Astrocyte Metabolism of the Medium‐Chain Fatty Acids Octanoic Acid and Decanoic Acid Promotes GABA Synthesis in Neurons via Elevated Glutamine Supply.” Molecular Brain 14: 132. 34479615 10.1186/s13041-021-00842-2 PMC8414667

[jnc70160-bib-0007] Anderzhanova, E. , T. Kirmeier , and C. T. Wotjak . 2017. “Animal Models in Psychiatric Research: The RDoC System as a New Framework for Endophenotype‐Oriented Translational Neuroscience.” Neurobiology of Stress 7: 47–56. 28377991 10.1016/j.ynstr.2017.03.003 PMC5377486

[jnc70160-bib-0008] Aper, S. J. A. , P. Dierickx , and M. Merkx . 2016. “Dual Readout BRET/FRET Sensors for Measuring Intracellular Zinc.” ACS Chemical Biology 11: 2854–2864. 27547982 10.1021/acschembio.6b00453 PMC5080634

[jnc70160-bib-0009] Arber, C. , A. Alatza , C. A. Leckey , R. W. Paterson , H. Zetterberg , and S. Wray . 2021. “Mass Spectrometry Analysis of Tau and Amyloid‐Beta in iPSC‐Derived Models of Alzheimer's Disease and Dementia.” Journal of Neurochemistry 159: 305–317. 33539581 10.1111/jnc.15315 PMC8613538

[jnc70160-bib-0010] Arizono, M. , A. Idziak , F. Quici , and U. V. Nägerl . 2023. “Getting Sharper: The Brain Under the Spotlight of Super‐Resolution Microscopy.” Trends in Cell Biology 33: 148–161. 35906123 10.1016/j.tcb.2022.06.011

[jnc70160-bib-0011] Armbruster, B. N. , X. Li , M. H. Pausch , S. Herlitze , and B. L. Roth . 2007. “Evolving the Lock to Fit the Key to Create a Family of G Protein‐Coupled Receptors Potently Activated by an Inert Ligand.” Proceedings of the National Academy of Sciences 104: 5163–5168. 10.1073/pnas.0700293104 PMC1829280 17360345

[jnc70160-bib-0012] Ashley, G. E. , T. Duong , M. T. Levenson , et al. 2021. “An Expanded Auxin‐Inducible Degron Toolkit for *Caenorhabditis elegans* .” Genetics 217: iyab006. 33677541 10.1093/genetics/iyab006 PMC8045686

[jnc70160-bib-0013] Atanas, A. A. , J. Kim , Z. Wang , et al. 2023. “Brain‐Wide Representations of Behavior Spanning Multiple Timescales and States in *C. elegans* .” Cell 186: 4134–4151.e31. 37607537 10.1016/j.cell.2023.07.035 PMC10836760

[jnc70160-bib-0014] Azkona, G. , and R. Sanchez‐Pernaute . 2022. “Mice in Translational Neuroscience: What R We Doing?” Progress in Neurobiology 217: 102330. 35872220 10.1016/j.pneurobio.2022.102330

[jnc70160-bib-0015] Badawi, Y. , and H. Nishimune . 2020. “Super‐Resolution Microscopy for Analyzing Neuromuscular Junctions and Synapses.” Neuroscience Letters 715: 134644. 31765730 10.1016/j.neulet.2019.134644 PMC6937598

[jnc70160-bib-0016] Baek, K. , K. Ji , W. Peng , S. M. Liyanaarachchi , and S. C. Dodani . 2021. “The Design and Evolution of Fluorescent Protein‐Based Sensors for Monoatomic Ions in Biology.” Protein Engineering, Design and Selection 34: gzab023. 10.1093/protein/gzab023 PMC8477612 34581820

[jnc70160-bib-0017] Bahr, B. A. 1995. “Long‐Term Hippocampal Slices: A Model System for Investigating Synaptic Mechanisms and Pathologic Processes.” Journal of Neuroscience Research 42: 294–305. 8583497 10.1002/jnr.490420303

[jnc70160-bib-0018] Bancelin, S. , L. Mercier , J. Roos , et al. 2023. “Imaging Dendritic Spines in the Hippocampus of a Living Mouse by 3D‐Stimulated Emission Depletion Microscopy.” Neurophotonics 10: 044402. 37215638 10.1117/1.NPh.10.4.044402 PMC10197143

[jnc70160-bib-0019] Bates, M. , B. Huang , G. T. Dempsey , and X. Zhuang . 2007. “Multicolor Super‐Resolution Imaging With Photo‐Switchable Fluorescent Probes.” Science 317: 1749–1753. 17702910 10.1126/science.1146598 PMC2633025

[jnc70160-bib-0020] Baxter, P. S. , O. Dando , K. Emelianova , et al. 2021. “Microglial Identity and Inflammatory Responses Are Controlled by the Combined Effects of Neurons and Astrocytes.” Cell Reports 34: 108882. 33761343 10.1016/j.celrep.2021.108882 PMC7994374

[jnc70160-bib-0021] Beauchamp, A. , Y. Yee , B. C. Darwin , A. Raznahan , R. B. Mars , and J. P. Lerch . 2022. “Whole‐Brain Comparison of Rodent and Human Brains Using Spatial Transcriptomics.” eLife 11: e79418. 36342372 10.7554/eLife.79418 PMC9708081

[jnc70160-bib-0022] Belmonte‐Mateos, C. , and C. Pujades . 2022. “From Cell States to Cell Fates: How Cell Proliferation and Neuronal Differentiation Are Coordinated During Embryonic Development.” Frontiers in Neuroscience 15: 781160. 35046768 10.3389/fnins.2021.781160 PMC8761814

[jnc70160-bib-0023] Bentzur, A. , S. Ben‐Shaanan , J. I. C. Benichou , et al. 2021. “Early Life Experience Shapes Male Behavior and Social Networks in *Drosophila* .” Current Biology 31: 486–501.e3. 33186552 10.1016/j.cub.2020.10.060

[jnc70160-bib-0024] Bernard, V. , P. Somogyi , and J. P. Bolam . 1997. “Cellular, Subcellular, and Subsynaptic Distribution of AMPA‐Type Glutamate Receptor Subunits in the Neostriatum of the Rat.” Journal of Neuroscience 17: 819–833. 8987803 10.1523/JNEUROSCI.17-02-00819.1997 PMC6573249

[jnc70160-bib-0025] Betzig, E. , G. H. Patterson , R. Sougrat , et al. 2006. “Imaging Intracellular Fluorescent Proteins at Nanometer Resolution.” Science 313: 1642–1645. 16902090 10.1126/science.1127344

[jnc70160-bib-0026] Białoń, M. , and A. Wąsik . 2022. “Advantages and Limitations of Animal Schizophrenia Models.” International Journal of Molecular Sciences 23: 5968. 35682647 10.3390/ijms23115968 PMC9181262

[jnc70160-bib-0027] Bianchi, F. , M. Malboubi , Y. Li , et al. 2018. “Rapid and Efficient Differentiation of Functional Motor Neurons From Human iPSC for Neural Injury Modelling.” Stem Cell Research 32: 126–134. 30278374 10.1016/j.scr.2018.09.006

[jnc70160-bib-0028] Birkmayer, W. , and O. Hornykiewicz . 1961. “The L‐3,4‐Dioxyphenylalanine (DOPA)‐Effect in Parkinson‐Akinesia.” Wiener Klinische Wochenschrift 73: 787–788. 13869404

[jnc70160-bib-0029] Birnbaum, J. H. , D. Wanner , A. F. Gietl , et al. 2018. “Oxidative Stress and Altered Mitochondrial Protein Expression in the Absence of Amyloid‐β and Tau Pathology in iPSC‐Derived Neurons From Sporadic Alzheimer's Disease Patients.” Stem Cell Research 27: 121–130. 29414602 10.1016/j.scr.2018.01.019

[jnc70160-bib-0030] Björklund, A. , and O. Lindvall . 2017. “Replacing Dopamine Neurons in Parkinson's Disease: How Did It Happen?” Journal of Parkinson's Disease 7: S21–S31. 10.3233/JPD-179002 PMC5345652 28282811

[jnc70160-bib-0031] Bohlen, C. J. , F. C. Bennett , and M. L. Bennett . 2019. “Isolation and Culture of Microglia.” Current Protocols in Immunology 125: e70. 30414379 10.1002/cpim.70 PMC6510657

[jnc70160-bib-0032] Boulin, T. , and O. Hobert . 2012. “From Genes to Function: The *C. elegans* Genetic Toolbox.” WIREs Developmental Biology 1: 114–137. 23801671 10.1002/wdev.1 PMC3694748

[jnc70160-bib-0033] Branda, C. S. , and M. J. Stern . 1999. “Cell Migration and Axon Growth Cone Guidance in *Caenorhabditis elegans* .” Current Opinion in Genetics & Development 9: 479–484. 10449355 10.1016/S0959-437X(99)80073-2

[jnc70160-bib-0034] Brunner, J. W. , H. C. A. Lammertse , A. A. van Berkel , et al. 2023. “Power and Optimal Study Design in iPSC‐Based Brain Disease Modelling.” Molecular Psychiatry 28: 1545–1556. 36385170 10.1038/s41380-022-01866-3 PMC10208961

[jnc70160-bib-0035] Bulaklak, K. , and C. A. Gersbach . 2020. “The Once and Future Gene Therapy.” Nature Communications 11: 5820. 10.1038/s41467-020-19505-2 PMC7670458 33199717

[jnc70160-bib-0036] Bulcha, J. T. , Y. Wang , H. Ma , P. W. L. Tai , and G. Gao . 2021. “Viral Vector Platforms Within the Gene Therapy Landscape.” Signal Transduction and Targeted Therapy 6: 1–24. 33558455 10.1038/s41392-021-00487-6 PMC7868676

[jnc70160-bib-0037] Cakir, B. , Y. Tanaka , F. R. Kiral , et al. 2022. “Expression of the Transcription Factor PU.1 Induces the Generation of Microglia‐Like Cells in Human Cortical Organoids.” Nature Communications 13: 430. 10.1038/s41467-022-28043-y PMC8776770 35058453

[jnc70160-bib-0038] Carter, M. , R. Essner , N. Goldstein , and M. Iyer . 2022. “Chapter 13—Cell Culture Techniques.” In Guide to Research Techniques in Neuroscience, edited by M. Carter , R. Essner , N. Goldstein , and M. Iyer , 3rd ed., 291–308. Academic Press.

[jnc70160-bib-0039] Cartier, N. , S. Hacein‐Bey‐Abina , C. C. Bartholomae , et al. 2009. “Hematopoietic Stem Cell Gene Therapy With a Lentiviral Vector in X‐Linked Adrenoleukodystrophy.” Science 326: 818–823. 19892975 10.1126/science.1171242

[jnc70160-bib-0040] Carvalhais, L. G. , V. C. Martinho , E. Ferreiro , and P. S. Pinheiro . 2021. “Unraveling the Nanoscopic Organization and Function of Central Mammalian Presynapses With Super‐Resolution Microscopy.” Frontiers in Neuroscience 14: 578409. 33584169 10.3389/fnins.2020.578409 PMC7874199

[jnc70160-bib-0041] Chadarevian, J. P. , S. I. Lombroso , G. C. Peet , et al. 2022. “Engineering an Inhibitor‐Resistant Human CSF1R Variant for Microglia Replacement.” Journal of Experimental Medicine 220: e20220857. 36584406 10.1084/jem.20220857 PMC9814156

[jnc70160-bib-0042] Cheepsunthorn, P. , L. Radov , S. Menzies , J. Reid , and J. R. Connor . 2001. “Characterization of a Novel Brain‐Derived Microglial Cell Line Isolated From Neonatal Rat Brain.” Glia 35: 53–62. 11424192 10.1002/glia.1070

[jnc70160-bib-0043] Chen, Y. , V. Balasubramaniyan , J. Peng , et al. 2007. “Isolation and Culture of Rat and Mouse Oligodendrocyte Precursor Cells.” Nature Protocols 2: 1044–1051. 17546009 10.1038/nprot.2007.149

[jnc70160-bib-0044] Chen, Y. , B. Stevens , J. Chang , J. Milbrandt , B. A. Barres , and J. W. Hell . 2008. “NS21: Re‐Defined and Modified Supplement B27 for Neuronal Cultures.” Journal of Neuroscience Methods 171: 239–247. 18471889 10.1016/j.jneumeth.2008.03.013 PMC2678682

[jnc70160-bib-0045] Ciccarelli, R. , P. Di Iorio , I. D'Alimonte , et al. 2000. “Cultured Astrocyte Proliferation Induced by Extracellular Guanosine Involves Endogenous Adenosine and Is Raised by the Co‐Presence of Microglia.” Glia 29: 202–211. 10642747

[jnc70160-bib-0046] Clark, S. , H. Jeong , A. Goehring , Y. Kang , and E. Gouaux . 2023. “Large‐Scale Growth of C. Elegans and Isolation of Membrane Protein Complexes.” Nature Protocols 18: 2699–2716. 37495753 10.1038/s41596-023-00852-5 PMC13012274

[jnc70160-bib-0047] Cook, S. J. , T. A. Jarrell , C. A. Brittin , et al. 2019. “Whole‐Animal Connectomes of Both *Caenorhabditis elegans* Sexes.” Nature 571: 63–71. 31270481 10.1038/s41586-019-1352-7 PMC6889226

[jnc70160-bib-0048] Counsell, J. R. , Z. Asgarian , J. Meng , et al. 2017. “Lentiviral Vectors Can Be Used for Full‐Length Dystrophin Gene Therapy.” Scientific Reports 7: 79. 28250438 10.1038/s41598-017-00152-5 PMC5427806

[jnc70160-bib-0049] Crain, J. M. , M. Nikodemova , and J. J. Watters . 2013. “Microglia Express Distinct M1 and M2 Phenotypic Markers in the Postnatal and Adult Central Nervous System in Male and Female Mice.” Journal of Neuroscience Research 91: 1143–1151. 23686747 10.1002/jnr.23242 PMC3715560

[jnc70160-bib-0050] Cristofari, P. , M. Desplanque , O. Poirel , et al. 2022. Nanoscopic Distribution of VAChT and VGLUT3 in Striatal Cholinergic Varicosities Suggests Colocalization and Segregation of the Two Transporters in Synaptic Vesicles. Vol. 15. Frontiers in Molecular Neuroscience. 10.3389/fnmol.2022.991732 PMC9513193 36176961

[jnc70160-bib-0051] Croft, C. L. , H. S. Futch , B. D. Moore , and T. E. Golde . 2019. “Organotypic Brain Slice Cultures to Model Neurodegenerative Proteinopathies.” Molecular Neurodegeneration 14: 45. 31791377 10.1186/s13024-019-0346-0 PMC6889333

[jnc70160-bib-0052] Cunningham, R. E. 2010. “Overview of Flow Cytometry and Fluorescent Probes for Flow Cytometry.” In Immunocytochemical Methods and Protocols, edited by C. Oliver and M. C. Jamur , 319–326. Humana Press.

[jnc70160-bib-0053] Das, A. , D. Fröhlich , L. B. Achanta , et al. 2020. “L‐Aspartate, L‐Ornithine and L‐Ornithine‐L‐Aspartate (LOLA) and Their Impact on Brain Energy Metabolism.” Neurochemical Research 45: 1438–1450. 32424601 10.1007/s11064-020-03044-9

[jnc70160-bib-0054] DeFelipe, J. 2011. “The Evolution of the Brain, the Human Nature of Cortical Circuits, and Intellectual Creativity.” Frontiers in Neuroanatomy 5: 29. 21647212 10.3389/fnana.2011.00029 PMC3098448

[jnc70160-bib-0055] Dello Russo, C. , N. Cappoli , I. Coletta , et al. 2018. “The Human Microglial HMC3 Cell Line: Where do We Stand? A Systematic Literature Review.” Journal of Neuroinflammation 15: 259. 30200996 10.1186/s12974-018-1288-0 PMC6131758

[jnc70160-bib-0056] Dempsey, G. T. , M. Bates , W. E. Kowtoniuk , D. R. Liu , R. Y. Tsien , and X. Zhuang . 2009. “Photoswitching Mechanism of Cyanine Dyes.” Journal of the American Chemical Society 131: 18192–18193. 19961226 10.1021/ja904588g PMC2797371

[jnc70160-bib-0057] Duff, K. , W. Noble , K. Gaynor , and Y. Matsuoka . 2002. “Organotypic Slice Cultures From Transgenic Mice as Disease Model Systems.” Journal of Molecular Neuroscience 19: 317–320. 12540058 10.1385/JMN:19:3:317

[jnc70160-bib-0058] Ehrlich, M. , S. Mozafari , M. Glatza , et al. 2017. “Rapid and Efficient Generation of Oligodendrocytes From Human Induced Pluripotent Stem Cells Using Transcription Factors.” Proceedings of the National Academy of Sciences 114: E2243–E2252. 10.1073/pnas.1614412114 PMC5358375 28246330

[jnc70160-bib-0059] El Mestikawy, S. , Å. Wallén‐Mackenzie , G. M. Fortin , L. Descarries , and L.‐E. Trudeau . 2011. “From Glutamate Co‐Release to Vesicular Synergy: Vesicular Glutamate Transporters.” Nature Reviews. Neuroscience 12: 204–216. 21415847 10.1038/nrn2969

[jnc70160-bib-0060] Elverum, K. , and M. Whitman . 2020. “Delivering Cellular and Gene Therapies to Patients: Solutions for Realizing the Potential of the Next Generation of Medicine.” Gene Therapy 27: 537–544. 31024072 10.1038/s41434-019-0074-7 PMC7744278

[jnc70160-bib-0061] Eugène, E. , F. Cluzeaud , C. Cifuentes‐Diaz , et al. 2014. “An Organotypic Brain Slice Preparation From Adult Patients With Temporal Lobe Epilepsy.” Journal of Neuroscience Methods 235: 234–244. 25064188 10.1016/j.jneumeth.2014.07.009 PMC4426207

[jnc70160-bib-0062] Fink, R. , S. Imai , N. Gockel , et al. 2025. “PinkyCaMP a mScarlet‐Based Calcium Sensor With Exceptional Brightness, Photostability, and Multiplexing Capabilities.” *bioRxiv*. 10.1038/s41592-026-03065-2 PMC13167472 42032066

[jnc70160-bib-0063] Flors, C. , J. Hotta , H. Uji‐i , et al. 2007. “A Stroboscopic Approach for Fast Photoactivation−Localization Microscopy With Dronpa Mutants.” Journal of the American Chemical Society 129: 13970–13977. 17956094 10.1021/ja074704l

[jnc70160-bib-0064] Franco, R. , and A. Cedazo‐Minguez . 2014. “Successful Therapies for Alzheimer's Disease: Why So Many in Animal Models and None in Humans?” Frontiers in Pharmacology 5: 146. 25009496 10.3389/fphar.2014.00146 PMC4070393

[jnc70160-bib-0065] Fuhrmann, M. , N. Gockel , M. Arizono , et al. 2022. “Super‐Resolution Microscopy Opens New Doors to Life at the Nanoscale.” Journal of Neuroscience 42: 8488–8497. 36351828 10.1523/JNEUROSCI.1125-22.2022 PMC9665916

[jnc70160-bib-0066] G. Anversa, R. , E. J. Campbell , L. C. Walker , et al. 2023. “A Paraventricular Thalamus to Insular Cortex Glutamatergic Projection Gates “Emotional” Stress‐Induced Binge Eating in Females.” Neuropsychopharmacology 48: 1931–1940. 37474763 10.1038/s41386-023-01665-6 PMC10584903

[jnc70160-bib-0067] Gähwiler, B. H. 1981. “Organotypic Monolayer Cultures of Nervous Tissue.” Journal of Neuroscience Methods 4: 329–342. 7033675 10.1016/0165-0270(81)90003-0

[jnc70160-bib-0068] Galatro, T. F. , I. R. Holtman , A. M. Lerario , et al. 2017. “Transcriptomic Analysis of Purified Human Cortical Microglia Reveals Age‐Associated Changes.” Nature Neuroscience 20: 1162–1171. 28671693 10.1038/nn.4597

[jnc70160-bib-0069] Galland, F. , M. Seady , J. Taday , S. S. Smaili , C. A. Gonçalves , and M. C. Leite . 2019. “Astrocyte Culture Models: Molecular and Function Characterization of Primary Culture, Immortalized Astrocytes and C6 Glioma Cells.” Neurochemistry International 131: 104538. 31430518 10.1016/j.neuint.2019.104538

[jnc70160-bib-0070] Gerrits, E. , Y. Heng , E. W. G. M. Boddeke , and B. J. L. Eggen . 2020. “Transcriptional Profiling of Microglia; Current State of the Art and Future Perspectives.” Glia 68: 740–755. 31846124 10.1002/glia.23767 PMC7064956

[jnc70160-bib-0071] Godin, A. G. , B. Lounis , and L. Cognet . 2014. “Super‐Resolution Microscopy Approaches for Live Cell Imaging.” Biophysical Journal 107: 1777–1784. 25418158 10.1016/j.bpj.2014.08.028 PMC4213717

[jnc70160-bib-0072] Goetz, C. G. 2011. “The History of Parkinson's Disease: Early Clinical Descriptions and Neurological Therapies.” Cold Spring Harbor Perspectives in Medicine 1: a008862. 22229124 10.1101/cshperspect.a008862 PMC3234454

[jnc70160-bib-0073] Gosai, S. J. , J. H. Kwak , C. J. Luke , et al. 2010. “Automated High‐Content Live Animal Drug Screening Using *C. elegans* Expressing the Aggregation Prone Serpin α1‐Antitrypsin Z.” PLoS One 5: e15460. 21103396 10.1371/journal.pone.0015460 PMC2980495

[jnc70160-bib-0074] Gourgou, E. , K. Adiga , A. Goettemoeller , C. Chen , and A.‐L. Hsu . 2021. “ *Caenorhabditis elegans* Learning in a Structured Maze Is a Multisensory Behavior.” iScience 24: 102284. 33889812 10.1016/j.isci.2021.102284 PMC8050377

[jnc70160-bib-0075] Gradisnik, L. , U. Maver , R. Bosnjak , and T. Velnar . 2020. “Optimised Isolation and Characterisation of Adult Human Astrocytes From Neurotrauma Patients.” Journal of Neuroscience Methods 341: 108796. 32450111 10.1016/j.jneumeth.2020.108796

[jnc70160-bib-0076] Griffin, E. F. , S. E. Scopel , C. A. Stephen , et al. 2019. “ApoE‐Associated Modulation of Neuroprotection From Aβ‐Mediated Neurodegeneration in Transgenic *Caenorhabditis elegans* .” Disease Models & Mechanisms 12: dmm037218. 30683808 10.1242/dmm.037218 PMC6398492

[jnc70160-bib-0077] Hanesch, U. , K.‐F. Fischbach , and M. Heisenberg . 1989. “Neuronal Architecture of the Central Complex in *Drosophila melanogaster* .” Cell and Tissue Research 257: 343–366. 10.1007/BF00327741 2124174

[jnc70160-bib-0078] Hara, A. , T. Taniguchi , T. Kanayama , and H. Tomita . 2024. “Immunohistochemistry of Brain Tissues.” In Cerebral Cortex Development: Methods and Protocols, edited by K. Nagata , 21–32. Springer US. 10.1007/978-1-0716-3810-1_3 38630217

[jnc70160-bib-0079] Harper, C. B. , and K. J. Smillie . 2021. “Current Molecular Approaches to Investigate Pre‐Synaptic Dysfunction.” Journal of Neurochemistry 157: 107–129. 33544872 10.1111/jnc.15316

[jnc70160-bib-0081] Hell, S. W. 2009. “Microscopy and Its Focal Switch.” Nature Methods 6: 24–32. 19116611 10.1038/nmeth.1291

[jnc70160-bib-0082] Hell, S. W. , and J. Wichmann . 1994. “Breaking the Diffraction Resolution Limit by Stimulated Emission: Stimulated‐Emission‐Depletion Fluorescence Microscopy.” Optics Letters 19: 780–782. 19844443 10.1364/ol.19.000780

[jnc70160-bib-0083] Hess, S. T. , T. P. K. Girirajan , and M. D. Mason . 2006. “Ultra‐High Resolution Imaging by Fluorescence Photoactivation Localization Microscopy.” Biophysical Journal 91: 4258–4272. 16980368 10.1529/biophysj.106.091116 PMC1635685

[jnc70160-bib-0084] Hess, S. T. , T. J. Gould , M. V. Gudheti , S. A. Maas , K. D. Mills , and J. Zimmerberg . 2007. “Dynamic Clustered Distribution of Hemagglutinin Resolved at 40 Nm in Living Cell Membranes Discriminates Between Raft Theories.” Proceedings of the National Academy of Sciences 104: 17370–17375. 10.1073/pnas.0708066104 PMC2077263 17959773

[jnc70160-bib-0085] Hoang, D. M. , P. T. Pham , T. Q. Bach , et al. 2022. “Stem Cell‐Based Therapy for Human Diseases.” Signal Transduction and Targeted Therapy 7: 1–41. 35933430 10.1038/s41392-022-01134-4 PMC9357075

[jnc70160-bib-0086] Hodge, R. D. , T. E. Bakken , J. A. Miller , et al. 2019. “Conserved Cell Types With Divergent Features in Human Versus Mouse Cortex.” Nature 573: 61–68. 31435019 10.1038/s41586-019-1506-7 PMC6919571

[jnc70160-bib-0087] Huang, B. , W. Wang , M. Bates , and X. Zhuang . 2008. “Three‐Dimensional Super‐Resolution Imaging by Stochastic Optical Reconstruction Microscopy.” Science 319: 810–813. 18174397 10.1126/science.1153529 PMC2633023

[jnc70160-bib-0088] Humpel, C. 2015. “Organotypic Brain Slice Cultures: A Review.” Neuroscience 305: 86–98. 26254240 10.1016/j.neuroscience.2015.07.086 PMC4699268

[jnc70160-bib-0089] Igarashi, M. , M. Nozumi , L.‐G. Wu , et al. 2018. “New Observations in Neuroscience Using Superresolution Microscopy.” Journal of Neuroscience 38: 9459–9467. 30381437 10.1523/JNEUROSCI.1678-18.2018 PMC6209844

[jnc70160-bib-0090] Jacquemet, G. , A. F. Carisey , H. Hamidi , R. Henriques , and C. Leterrier . 2020. “The Cell Biologist's Guide to Super‐Resolution Microscopy.” Journal of Cell Science 133: jcs240713. 32527967 10.1242/jcs.240713

[jnc70160-bib-0091] Jagadisan, B. , and A. Dhawan . 2023. “Hepatotoxicity in Adeno‐Associated Viral Vector Gene Therapy.” Current Hepatology Reports 22: 276–290.

[jnc70160-bib-0092] Jones, R. A. , C. Harrison , S. L. Eaton , et al. 2017. “Cellular and Molecular Anatomy of the Human Neuromuscular Junction.” Cell Reports 21: 2348–2356. 29186674 10.1016/j.celrep.2017.11.008 PMC5723673

[jnc70160-bib-0093] Jordan, E. T. , M. Collins , J. Terefe , L. Ugozzoli , and T. Rubio . 2008. “Optimizing Electroporation Conditions in Primary and Other Difficult‐To‐Transfect Cells.” Journal of Biomolecular Techniques 19: 328–334. 19183796 PMC2628074

[jnc70160-bib-0094] Jungmann, R. , M. S. Avendaño , J. B. Woehrstein , M. Dai , W. M. Shih , and P. Yin . 2014. “Multiplexed 3D Cellular Super‐Resolution Imaging With DNA‐PAINT and Exchange‐PAINT.” Nature Methods 11: 313–318. 24487583 10.1038/nmeth.2835 PMC4153392

[jnc70160-bib-0095] Kaiser, R. A. , C. T. Nicolas , K. L. Allen , et al. 2019. “Hepatotoxicity and Toxicology of In Vivo Lentiviral Vector Administration in Healthy and Liver‐Injury Mouse Models.” Human Gene Therapy Clinical Development 30: 57–66. 30860398 10.1089/humc.2018.249 PMC6589498

[jnc70160-bib-0096] Kalyuzhny, A. E. , ed. 2011. “Signal Transduction Immunohistochemistry: Methods and Protocols.” In Methods in Molecular Biology, vol. 717. Humana Press.

[jnc70160-bib-0097] Kater, M. S. J. , A. Badia‐Soteras , J. R. T. Weering , A. B. van, Smit , and M. H. G. Verheijen . 2023. “Electron Microscopy Analysis of Astrocyte‐Synapse Interactions Shows Altered Dynamics in an Alzheimer's Disease Mouse Model.” Frontiers in Cellular Neuroscience 17: 1085690. 36779013 10.3389/fncel.2023.1085690 PMC9908992

[jnc70160-bib-0098] Kavalali, E. T. , and E. M. Jorgensen . 2014. “Visualizing Presynaptic Function.” Nature Neuroscience 17: 10–16. 24369372 10.1038/nn.3578

[jnc70160-bib-0099] Kim, D.‐S. , D. R. Lee , H.‐S. Kim , et al. 2012. “Highly Pure and Expandable PSA‐NCAM‐Positive Neural Precursors From Human ESC and iPSC‐Derived Neural Rosettes.” PLoS One 7: e39715. 22911689 10.1371/journal.pone.0039715 PMC3401209

[jnc70160-bib-0100] Kim, M.‐H. , C. Radaelli , E. R. Thomsen , et al. 2023. “Target Cell‐Specific Synaptic Dynamics of Excitatory to Inhibitory Neuron Connections in Supragranular Layers of Human Neocortex.” eLife 12: e81863. 37249212 10.7554/eLife.81863 PMC10332811

[jnc70160-bib-0101] Kim, Y. , Y. Park , J. Hwang , and K. Kwack . 2018. “Comparative Genomic Analysis of the Human and Nematode *Caenorhabditis elegans* Uncovers Potential Reproductive Genes and Disease Associations in Humans.” Physiological Genomics 50: 1002–1014. 30240344 10.1152/physiolgenomics.00063.2018

[jnc70160-bib-0102] King, C. E. , W. C. Griffin , M. F. Lopez , and H. C. Becker . 2021. “Activation of Hypothalamic Oxytocin Neurons Reduces Binge‐Like Alcohol Drinking Through Signaling at Central Oxytocin Receptors.” Neuropsychopharmacology 46: 1950–1957. 34127796 10.1038/s41386-021-01046-x PMC8429589

[jnc70160-bib-0104] Konstantinidis, E. , A. Molisak , F. Perrin , et al. 2022. “CRISPR‐Cas9 Treatment Partially Restores Amyloid‐β 42/40 in Human Fibroblasts With the Alzheimer's Disease PSEN1 M146L Mutation.” Molecular Therapy Nucleic Acids 28: 450–461. 35505961 10.1016/j.omtn.2022.03.022 PMC9043867

[jnc70160-bib-0105] Kostiuk, G. , J. Bucevičius , R. Gerasimaitė , and G. Lukinavičius . 2019. “Application of STED Imaging for Chromatin Studies.” Journal of Physics D: Applied Physics 52: 504003.

[jnc70160-bib-0106] Kovalevich, J. , and D. Langford . 2013. “Considerations for the Use of SH‐SY5Y Neuroblastoma Cells in Neurobiology.” In Neuronal Cell Culture: Methods and Protocols, edited by S. Amini and M. K. White , 9–21. Humana Press. 10.1007/978-1-62703-640-5_2 PMC5127451 23975817

[jnc70160-bib-0107] Kravitz, E. A. , and M. D. L. P. Fernandez . 2015. “Aggression in Drosophila.” Behavioral Neuroscience 129: 549–563. 26348714 10.1037/bne0000089

[jnc70160-bib-0108] Kulshreshtha, A. , and A. Agrawal . 2020. “Chapter 10 ‐ Regulation by Non‐Coding RNAs in Respiratory Disorders.” In Rna‐Based Regulation in Human Health and Disease, edited by R. Pandey , vol. 19, 233–249. Academic Press.

[jnc70160-bib-0109] Kutscher, L. M. , and S. Shaham . 2014. “Forward and Reverse Mutagenesis in *C. elegans* .” WormBook 17: 1–26. 10.1895/wormbook.1.167.1 PMC4078664 24449699

[jnc70160-bib-0110] Kwon, J. , J. Hwang , J. Park , G. R. Han , K. Y. Han , and S. K. Kim . 2015. “RESOLFT Nanoscopy With Photoswitchable Organic Fluorophores.” Scientific Reports 5: 17804. 26639557 10.1038/srep17804 PMC4671063

[jnc70160-bib-0111] Lelek, M. , M. T. Gyparaki , G. Beliu , et al. 2021. “Single‐Molecule Localization Microscopy.” Nature Reviews Methods Primers 1: 1–27. 10.1038/s43586-021-00038-x PMC9160414 35663461

[jnc70160-bib-0112] Lendemeijer, B. , M. Unkel , H. Smeenk , et al. 2024. “Human Pluripotent Stem Cell‐Derived Astrocyte Functionality Compares Favorably With Primary Rat Astrocytes.” eNeuro 11. 10.1523/ENEURO.0148-24.2024 PMC11404293 39227152

[jnc70160-bib-0113] Lin, S. , Y. Lin , J. R. Nery , et al. 2014. “Comparison of the Transcriptional Landscapes Between Human and Mouse Tissues.” Proceedings of the National Academy of Sciences of the United States of America 111: 17224–17229. 25413365 10.1073/pnas.1413624111 PMC4260565

[jnc70160-bib-0114] Lin, Y.‐T. , J. Seo , F. Gao , et al. 2018. “ *APOE4* Causes Widespread Molecular and Cellular Alterations Associated With Alzheimer's Disease Phenotypes in Human iPSC‐Derived Brain Cell Types.” Neuron 98: 1141–1154.e7. 29861287 10.1016/j.neuron.2018.05.008 PMC6023751

[jnc70160-bib-0115] Lindsay, T. H. , T. R. Thiele , and S. R. Lockery . 2011. “Optogenetic Analysis of Synaptic Transmission in the Central Nervous System of the Nematode *Caenorhabditis elegans* .” Nature Communications 2: 306. 10.1038/ncomms1304 PMC3935721 21556060

[jnc70160-bib-0116] Lippincott‐Schwartz, J. , and G. H. Patterson . 2009. “Photoactivatable Fluorescent Proteins for Diffraction‐Limited and Super‐Resolution Imaging.” Trends in Cell Biology 19: 555–565. 19836954 10.1016/j.tcb.2009.09.003 PMC3663713

[jnc70160-bib-0117] Liu, S. , P. Hoess , and J. Ries . 2022. “Super‐Resolution Microscopy for Structural Cell Biology.” Annual Review of Biophysics 51: 301–326. 10.1146/annurev-biophys-102521-112912 35119945

[jnc70160-bib-0118] Loomba, S. , J. Straehle , V. Gangadharan , et al. 2022. “Connectomic Comparison of Mouse and Human Cortex.” Science 377: eabo0924. 35737810 10.1126/science.abo0924

[jnc70160-bib-0119] Louis, J. C. , E. Magal , D. Muir , M. Manthorpe , and S. Varon . 1992. “CG‐4, A New Bipotential Glial Cell Line From Rat Brain, Is Capable of Differentiating In Vitro Into Either Mature Oligodendrocytes or Type‐2 Astrocytes.” Journal of Neuroscience Research 31: 193–204. 1613821 10.1002/jnr.490310125

[jnc70160-bib-0120] Lyman, W. D. , M. Tricoche , W. C. Hatch , Y. Kress , F. C. Chiu , and W. K. Rashbaum . 1991. “Human Fetal Central Nervous System Organotypic Cultures.” Brain Research. Developmental Brain Research 60: 155–160. 1893565 10.1016/0165-3806(91)90044-j

[jnc70160-bib-0121] Machiela, E. , P. D. Rudich , A. Traa , et al. 2021. “Targeting Mitochondrial Network Disorganization Is Protective in *C. elegans* Models of Huntington's Disease.” Aging and Disease 12: 1753–1772. 34631219 10.14336/AD.2021.0404 PMC8460302

[jnc70160-bib-0123] Manger, P. , J. Cort , N. Ebrahim , et al. 2008. “Is 21st Century Neuroscience Too Focussed on the Rat/Mouse Model of Brain Function and Dysfunction?” Frontiers in Neuroanatomy 2: 5. 19127284 10.3389/neuro.05.005.2008 PMC2605402

[jnc70160-bib-0124] Manz, K. M. , J. K. Siemann , D. G. McMahon , and B. A. Grueter . 2021. “Patch‐Clamp and Multi‐Electrode Array Electrophysiological Analysis in Acute Mouse Brain Slices.” STAR Protocols 2: 100442. 33899023 10.1016/j.xpro.2021.100442 PMC8056272

[jnc70160-bib-0125] Martin‐Fernandez, M. , S. Jamison , L. M. Robin , et al. 2017. “Synapse‐Specific Astrocyte Gating of Amygdala‐Related Behavior.” Nature Neuroscience 20: 1540–1548. 28945222 10.1038/nn.4649 PMC5903286

[jnc70160-bib-0126] Massengill, C. I. , J. Day‐Cooney , T. Mao , and H. Zhong . 2021. “Genetically Encoded Sensors Towards Imaging cAMP and PKA Activity In Vivo.” Journal of Neuroscience Methods 362: 109298. 34339753 10.1016/j.jneumeth.2021.109298 PMC8659126

[jnc70160-bib-0127] Miesenböck, G. , D. A. De Angelis , and J. E. Rothman . 1998. “Visualizing Secretion and Synaptic Transmission With pH‐Sensitive Green Fluorescent Proteins.” Nature 394: 192–195. 9671304 10.1038/28190

[jnc70160-bib-0128] Miyawaki, A. , J. Llopis , R. Heim , et al. 1997. “Fluorescent Indicators for Ca2+Based on Green Fluorescent Proteins and Calmodulin.” Nature 388: 882–887. 9278050 10.1038/42264

[jnc70160-bib-0129] Mizumoto, K. , Y. Jin , and J.‐L. Bessereau . 2023. “Synaptogenesis: Unmasking Molecular Mechanisms Using *Caenorhabditis elegans* .” Genetics 223: iyac176. 36630525 10.1093/genetics/iyac176 PMC9910414

[jnc70160-bib-0130] Mollá‐Albaladejo, R. , and J. A. Sánchez‐Alcañiz . 2021. “Behavior Individuality: A Focus on *Drosophila melanogaster* .” Frontiers in Physiology 12: 719038. 34916952 10.3389/fphys.2021.719038 PMC8670942

[jnc70160-bib-0131] Molnár, Z. , and G. Clowry . 2012. “Chapter 3—Cerebral Cortical Development in Rodents and Primates.” In Progress in Brain Research, edited by M. A. Hofman and D. Falk , vol. 195, 45–70. Elsevier. 10.1016/B978-0-444-53860-4.00003-9 22230622

[jnc70160-bib-0132] Mondal, S. , E. Hegarty , C. Martin , S. K. Gökçe , N. Ghorashian , and A. Ben‐Yakar . 2016. “Large‐Scale Microfluidics Providing High‐Resolution and High‐Throughput Screening of *Caenorhabditis elegans* Poly‐Glutamine Aggregation Model.” Nature Communications 7: 13023. 10.1038/ncomms13023 PMC5062571 27725672

[jnc70160-bib-0133] Nägerl, U. V. , K. I. Willig , B. Hein , S. W. Hell , and T. Bonhoeffer . 2008. “Live‐Cell Imaging of Dendritic Spines by STED Microscopy.” Proceedings of the National Academy of Sciences 105: 18982–18987. 10.1073/pnas.0810028105 PMC2585941 19028874

[jnc70160-bib-0134] Nance, J. , and C. Frøkjær‐Jensen . 2019. “The *Caenorhabditis elegans* Transgenic Toolbox.” Genetics 212: 959–990. 31405997 10.1534/genetics.119.301506 PMC6707460

[jnc70160-bib-0135] Oakley, D. H. , N. Klickstein , C. Commins , et al. 2021. “Continuous Monitoring of Tau‐Induced Neurotoxicity in Patient‐Derived iPSC‐Neurons.” Journal of Neuroscience 41: 4335–4348. 33893219 10.1523/JNEUROSCI.2590-20.2021 PMC8143197

[jnc70160-bib-0136] Oikonomou, G. , and S. Shaham . 2011. “The Glia of *Caenorhabditis elegans* .” Glia 59: 1253–1263. 21732423 10.1002/glia.21084 PMC3117073

[jnc70160-bib-0137] Pamies, D. 2021. Guidance Document on Good Cell and Tissue Culture Practice 2.0 (GCCP 2.0). ALTEX. 10.14573/altex.2111011 34882777

[jnc70160-bib-0138] Pandey, U. B. , and C. D. Nichols . 2011. “Human Disease Models in Drosophila Melanogaster and the Role of the Fly in Therapeutic Drug Discovery.” Drug Delivery 63: 411–436. 10.1124/pr.110.003293 PMC3082451 21415126

[jnc70160-bib-0139] Papassotiropoulos, A. , J. Petrovska , A. Arnold , et al. 2024. “Identification of a Unique, De Novo MYCBP2 Variant in an Individual With Highly Superior Autobiographical Memory.” *medRxiv*.

[jnc70160-bib-0140] Park, T. I.‐H. , P. Schweder , K. Lee , et al. 2020. “Isolation and Culture of Functional Adult Human Neurons From Neurosurgical Brain Specimens.” Brain Communications 2: fcaa171. 33215086 10.1093/braincomms/fcaa171 PMC7660143

[jnc70160-bib-0141] Patra, P. H. , B. Tench , T. Hitrec , et al. 2023. “Pro‐Opiomelanocortin Neurons in the Nucleus of the Solitary Tract Mediate Endorphinergic Endogenous Analgesia in Mice.” Pain 164: 1051–1066. 36448978 10.1097/j.pain.0000000000002802

[jnc70160-bib-0142] Patterson, G. H. , and J. Lippincott‐Schwartz . 2002. “A Photoactivatable GFP for Selective Photolabeling of Proteins and Cells.” Science 297: 1873–1877. 12228718 10.1126/science.1074952

[jnc70160-bib-0143] Penczek, P. A. 2010. “Chapter Three ‐ Resolution Measures in Molecular Electron Microscopy.” In Methods in Enzymology, edited by G. J. Jensen , vol. 482, 73–100. Academic Press. 20888958 10.1016/S0076-6879(10)82003-8 PMC3165049

[jnc70160-bib-0144] Pennacchietti, F. , S. Vascon , T. Nieus , et al. 2017. “Nanoscale Molecular Reorganization of the Inhibitory Postsynaptic Density Is a Determinant of GABAergic Synaptic Potentiation.” Journal of Neuroscience 37: 1747–1756. 28073939 10.1523/JNEUROSCI.0514-16.2016 PMC6589977

[jnc70160-bib-0145] Petrascheck, M. , X. Ye , and L. B. Buck . 2007. “An Antidepressant That Extends Lifespan in Adult *Caenorhabditis elegans* .” Nature 450: 553–556. 18033297 10.1038/nature05991

[jnc70160-bib-0146] Pfeiffer, R. , and M. Ebadi . 1972. “On the Mechanism of the Nullification of CNS Effects of L‐Dopa by Pyridoxine in Parkinsonian Patients.” Journal of Neurochemistry 19: 2175–2181. 5072392 10.1111/j.1471-4159.1972.tb05126.x

[jnc70160-bib-0147] Qi, G. , G. Radnikow , and D. Feldmeyer . 2015. “Electrophysiological and Morphological Characterization of Neuronal Microcircuits in Acute Brain Slices Using Paired Patch‐Clamp Recordings.” JoVE 95: 52358. 10.3791/52358 PMC4354532 25650985

[jnc70160-bib-0148] Rakic, P. 2009. “Evolution of the Neocortex: A Perspective From Developmental Biology.” Nature Reviews. Neuroscience 10: 724–735. 19763105 10.1038/nrn2719 PMC2913577

[jnc70160-bib-0149] Ranganathan, R. , E. R. Sawin , C. Trent , and H. R. Horvitz . 2001. “Mutations in the *Caenorhabditis elegans* Serotonin Reuptake Transporter MOD‐5 Reveal Serotonin‐Dependent and ‐Independent Activities of Fluoxetine.” Journal of Neuroscience 21: 5871–5884. 11487610 10.1523/JNEUROSCI.21-16-05871.2001 PMC6763176

[jnc70160-bib-0150] Rasti, A. R. , V. E. Coombe , J. R. Muzik , and C. L. Kliethermes . 2020. “Pharmacological Characterization of the Forced Swim Test in *Drosophila melanogaster* .” Invertebrate Neuroscience 20: 22. 33170389 10.1007/s10158-020-00255-1

[jnc70160-bib-0151] Rinken, A. , D. Lavogina , and S. Kopanchuk . 2018. “Assays With Detection of Fluorescence Anisotropy: Challenges and Possibilities for Characterizing Ligand Binding to GPCRs.” Trends in Pharmacological Sciences 39: 187–199. 29102621 10.1016/j.tips.2017.10.004

[jnc70160-bib-0152] Rohner, V. L. , P. J. Lamothe‐Molina , and T. Patriarchi . 2024. “Engineering, Applications, and Future Perspectives of GPCR‐Based Genetically Encoded Fluorescent Indicators for Neuromodulators.” Journal of Neurochemistry 168: 163–184. 38288673 10.1111/jnc.16045

[jnc70160-bib-0153] Rooke, R. , A. Rasool , J. Schneider , and J. D. Levine . 2020. “ *Drosophila melanogaster* Behaviour Changes in Different Social Environments Based on Group Size and Density.” Communications Biology 3: 1–6. 32533063 10.1038/s42003-020-1024-z PMC7293324

[jnc70160-bib-0155] Rutherford, M. A. 2015. “Resolving the Structure of Inner Ear Ribbon Synapses With STED Microscopy.” Synapse 69: 242–255. 25682928 10.1002/syn.21812

[jnc70160-bib-0156] Sahu, M. P. , O. Nikkilä , S. Lågas , S. Kolehmainen , and E. Castrén . 2019. “Culturing Primary Neurons From Rat Hippocampus and Cortex.” Neuronal Signaling 3: NS20180207. 32714598 10.1042/NS20180207 PMC7363313

[jnc70160-bib-0157] Sánchez, D. J. L.‐D. , F. W. Lindhout , A. J. Anderson , L. Pellegrini , and M. A. Lancaster . 2024. “Mouse Brain Organoids Model In Vivo Neurodevelopment and Function and Capture Differences to Human.” *bioRxiv*.

[jnc70160-bib-0158] Sapir, G. , D. Shaul , N. Lev‐Cohain , J. Sosna , M. J. Gomori , and R. Katz‐Brull . 2021. “LDH and PDH Activities in the Ischemic Brain and the Effect of Reperfusion—An Ex Vivo MR Study in Rat Brain Slices Using Hyperpolarized [1‐13C]Pyruvate.” Metabolites 11: 210. 33808434 10.3390/metabo11040210 PMC8066106

[jnc70160-bib-0159] Sapkal, N. , N. Mancini , D. S. Kumar , et al. 2024. “Neural Circuit Mechanisms Underlying Context‐Specific Halting in Drosophila.” Nature 634: 191–200. 39358520 10.1038/s41586-024-07854-7 PMC11446846

[jnc70160-bib-0160] Sasaki, T. , I. Suzuki , R. Yokoi , K. Sato , and Y. Ikegaya . 2019. “Synchronous Spike Patterns in Differently Mixed Cultures of Human iPSC‐Derived Glutamatergic and GABAergic Neurons.” Biochemical and Biophysical Research Communications 513: 300–305. 30954214 10.1016/j.bbrc.2019.03.161

[jnc70160-bib-0161] Schermelleh, L. , A. Ferrand , T. Huser , et al. 2019. “Super‐Resolution Microscopy Demystified.” Nature Cell Biology 21: 72–84. 30602772 10.1038/s41556-018-0251-8

[jnc70160-bib-0162] Schildge, S. , C. Bohrer , K. Beck , C. Schachtrup , S. Schildge , and C. Bohrer . 2013. “Isolation and Culture of Mouse Cortical Astrocytes.” Journal of Visualized Experiments 71: e50079. 10.3791/50079 PMC3582677 23380713

[jnc70160-bib-0163] Schwartzentruber, J. , S. Foskolou , H. Kilpinen , et al. 2018. “Molecular and Functional Variation in iPSC‐Derived Sensory Neurons.” Nature Genetics 50: 54–61. 29229984 10.1038/s41588-017-0005-8 PMC5742539

[jnc70160-bib-0164] Schwarz, N. , U. B. S. Hedrich , H. Schwarz , et al. 2017. “Human Cerebrospinal Fluid Promotes Long‐Term Neuronal Viability and Network Function in Human Neocortical Organotypic Brain Slice Cultures.” Scientific Reports 7: 12249. 28947761 10.1038/s41598-017-12527-9 PMC5613008

[jnc70160-bib-0165] Sharonov, A. , and R. M. Hochstrasser . 2006. “Wide‐Field Subdiffraction Imaging by Accumulated Binding of Diffusing Probes.” Proceedings of the National Academy of Sciences 103: 18911–18916. 10.1073/pnas.0609643104 PMC1748151 17142314

[jnc70160-bib-0166] Simonetta, S. H. , and D. A. Golombek . 2007. “An Automated Tracking System for *Caenorhabditis elegans* Locomotor Behavior and Circadian Studies Application.” Journal of Neuroscience Methods 161: 273–280. 17207862 10.1016/j.jneumeth.2006.11.015

[jnc70160-bib-0167] Sitaraman, D. , M. Zars , H. LaFerriere , et al. 2008. “Serotonin Is Necessary for Place Memory in Drosophila.” Proceedings of the National Academy of Sciences 105: 5579–5584. 10.1073/pnas.0710168105 PMC2291120 18385379

[jnc70160-bib-0168] Smart, I. H. M. , C. Dehay , P. Giroud , M. Berland , and H. Kennedy . 2002. “Unique Morphological Features of the Proliferative Zones and Postmitotic Compartments of the Neural Epithelium Giving Rise to Striate and Extrastriate Cortex in the Monkey.” Cerebral Cortex 12: 37–53. 11734531 10.1093/cercor/12.1.37 PMC1931430

[jnc70160-bib-0169] Snyder, J. S. , J. S. Choe , M. A. Clifford , et al. 2009. “Adult‐Born Hippocampal Neurons Are More Numerous, Faster Maturing, and More Involved in Behavior in Rats Than in Mice.” Journal of Neuroscience 29: 14484–14495. 19923282 10.1523/JNEUROSCI.1768-09.2009 PMC2830901

[jnc70160-bib-0170] Sohrabi, S. , D. E. Mor , R. Kaletsky , W. Keyes , and C. T. Murphy . 2021. “High‐Throughput Behavioral Screen in *C. elegans* Reveals Parkinson's Disease Drug Candidates.” Communications Biology 4: 1–9. 33589689 10.1038/s42003-021-01731-z PMC7884385

[jnc70160-bib-0171] Soice, E. , and J. Johnston . 2021. “Immortalizing Cells for Human Consumption.” International Journal of Molecular Sciences 22: 11660. 34769088 10.3390/ijms222111660 PMC8584139

[jnc70160-bib-0172] Stansley, B. , J. Post , and K. Hensley . 2012. “A Comparative Review of Cell Culture Systems for the Study of Microglial Biology in Alzheimer's Disease.” Journal of Neuroinflammation 9: 115. 22651808 10.1186/1742-2094-9-115 PMC3407712

[jnc70160-bib-0173] Stoddart, L. A. , L. E. Kilpatrick , and S. J. Hill . 2018. “NanoBRET Approaches to Study Ligand Binding to GPCRs and RTKs.” Trends in Pharmacological Sciences 39: 136–147. 29132917 10.1016/j.tips.2017.10.006

[jnc70160-bib-0174] Stoppini, L. , P. A. Buchs , and D. Muller . 1991. “A Simple Method for Organotypic Cultures of Nervous Tissue.” Journal of Neuroscience Methods 37: 173–182. 1715499 10.1016/0165-0270(91)90128-m

[jnc70160-bib-0175] Stroustrup, N. , B. E. Ulmschneider , Z. M. Nash , I. F. López‐Moyado , J. Apfeld , and W. Fontana . 2013. “The *Caenorhabditis elegans* Lifespan Machine.” Nature Methods 10: 665–670. 23666410 10.1038/nmeth.2475 PMC3865717

[jnc70160-bib-0176] Sugie, A. , G. Marchetti , and G. Tavosanis . 2018. “Structural Aspects of Plasticity in the Nervous System of Drosophila.” Neural Development 13: 14. 29960596 10.1186/s13064-018-0111-z PMC6026517

[jnc70160-bib-0177] Svensson, E. , J. Apergis‐Schoute , G. Burnstock , M. P. Nusbaum , D. Parker , and H. B. Schiöth . 2019. “General Principles of Neuronal co‐Transmission: Insights From Multiple Model Systems.” Frontiers in Neural Circuits 12: 117. 30728768 10.3389/fncir.2018.00117 PMC6352749

[jnc70160-bib-0178] Szabó, R. , Á. Hornyánszky , D. J. Kiss , and G. M. Keserű . 2025. “Fluorescent Tools for Imaging Class A G‐Protein Coupled Receptors.” European Journal of Pharmaceutical Sciences 209: 107074. 40113106 10.1016/j.ejps.2025.107074

[jnc70160-bib-0179] Teixeira‐Castro, A. , J. C. Sousa , C. Vieira , et al. 2023. “Learning the Biochemical Basis of Axonal Guidance: Using *Caenorhabditis elegans* as a Model.” Biomedicine 11: 1731. 10.3390/biomedicines11061731 PMC10296117 37371826

[jnc70160-bib-0180] Tuijtel, M. W. , A. J. Koster , S. Jakobs , F. G. A. Faas , and T. H. Sharp . 2019. “Correlative Cryo Super‐Resolution Light and Electron Microscopy on Mammalian Cells Using Fluorescent Proteins.” Scientific Reports 9: 1369. 30718653 10.1038/s41598-018-37728-8 PMC6362030

[jnc70160-bib-0181] Uytterhoeven, V. , S. Kuenen , J. Kasprowicz , K. Miskiewicz , and P. Verstreken . 2011. “Loss of Skywalker Reveals Synaptic Endosomes as Sorting Stations for Synaptic Vesicle Proteins.” Cell 145: 117–132. 21458671 10.1016/j.cell.2011.02.039

[jnc70160-bib-0182] Vaaga, C. E. , M. Borisovska , and G. L. Westbrook . 2014. “Dual‐Transmitter Neurons: Functional Implications of Co‐Release and Co‐Transmission.” Current Opinion in Neurobiology 29: 25–32. 24816154 10.1016/j.conb.2014.04.010 PMC4231002

[jnc70160-bib-0184] van der Kant, R. , V. F. Langness , C. M. Herrera , et al. 2019. “Cholesterol Metabolism Is a Druggable Axis That Independently Regulates Tau and Amyloid‐β in iPSC‐Derived Alzheimer's Disease Neurons.” Cell Stem Cell 24: 363–375.e9. 30686764 10.1016/j.stem.2018.12.013 PMC6414424

[jnc70160-bib-0185] van der Valk, J. , D. Brunner , K. De Smet , et al. 2010. “Optimization of Chemically Defined Cell Culture Media—Replacing Fetal Bovine Serum in Mammalian In Vitro Methods.” Toxicology In Vitro 24: 1053–1063. 20362047 10.1016/j.tiv.2010.03.016

[jnc70160-bib-0187] Verstreken, P. , T. Ohyama , C. Haueter , et al. 2009. “Tweek, an Evolutionarily Conserved Protein, Is Required for Synaptic Vesicle Recycling.” Neuron 63: 203–215. 19640479 10.1016/j.neuron.2009.06.017 PMC2759194

[jnc70160-bib-0188] Verwer, R. W. H. , R. E. Baker , E. F. M. Boiten , et al. 2003. “Post‐Mortem Brain Tissue Cultures From Elderly Control Subjects and Patients With a Neurodegenerative Disease.” Experimental Gerontology 38: 167–172. 12543274 10.1016/s0531-5565(02)00154-7

[jnc70160-bib-0189] Vijaya, A. K. , M. Iešmantaitė , V. Mela , D. Baltriukienė , and A. Burokas . 2023. “Microglia Isolation From Aging Mice for Cell Culture: A Beginner's Guide.” Frontiers in Cellular Neuroscience 17: 1082180. 36744004 10.3389/fncel.2023.1082180 PMC9893793

[jnc70160-bib-0190] Vlasov, K. , C. J. Van Dort , and K. Solt . 2018. “Optogenetics and Chemogenetics.” Methods in Enzymology 603: 181–196. 29673525 10.1016/bs.mie.2018.01.022 PMC6984397

[jnc70160-bib-0191] Wang, C. , M. E. Ward , R. Chen , et al. 2017. “Scalable Production of iPSC‐Derived Human Neurons to Identify Tau‐Lowering Compounds by High‐Content Screening.” Stem Cell Reports 9: 1221–1233. 28966121 10.1016/j.stemcr.2017.08.019 PMC5639430

[jnc70160-bib-0192] Wang, S. , N. H. Tang , P. Lara‐Gonzalez , et al. 2017. “A Toolkit for GFP‐Mediated Tissue‐Specific Protein Degradation in *C. elegans* .” Development 144: 2694–2701. 28619826 10.1242/dev.150094 PMC5536931

[jnc70160-bib-0193] Wenker, I. C. , A. R. Boscia , C. Lewis , et al. 2022. “Forebrain Epileptiform Activity Is Not Required for Seizure‐Induced Apnea in a Mouse Model of Scn8a Epilepsy.” Frontiers in Neural Circuits 16: 1002013. 36160949 10.3389/fncir.2022.1002013 PMC9490431

[jnc70160-bib-0194] Wenzel, T. J. , J. Le , J. He , J. Alcorn , and D. D. Mousseau . 2023. “Fundamental Neurochemistry Review: Incorporating a Greater Diversity of Cell Types, Including Microglia, in Brain Organoid Cultures Improves Clinical Translation.” Journal of Neurochemistry 164: 560–582. 36517959 10.1111/jnc.15741

[jnc70160-bib-0196] Wenzel, T. J. , and D. D. Mousseau . 2025. “16—Maximizing the Utility of Brain Organoid Models and Overcoming Their Perceived Limitations.” In Handbook of Neural Engineering, edited by S. Willerth , 593–624. Academic Press.

[jnc70160-bib-0197] Wester, L. E. , A. Lanjuin , E. H. W. Bruckisch , et al. 2023. “A Single‐Copy Knockin Translating Ribosome Immunoprecipitation Toolkit for Tissue‐Specific Profiling of Actively Translated mRNAs in *C. elegans* .” Cell Reports Methods 3: 100433. 37056370 10.1016/j.crmeth.2023.100433 PMC10088236

[jnc70160-bib-0198] Westphal, V. , S. O. Rizzoli , M. A. Lauterbach , D. Kamin , R. Jahn , and S. W. Hell . 2008. “Video‐Rate Far‐Field Optical Nanoscopy Dissects Synaptic Vesicle Movement.” Science 320: 246–249. 18292304 10.1126/science.1154228

[jnc70160-bib-0199] Wong, H. H.‐W. , C. Y. C. Chou , A. J. Watt , and P. J. Sjöström . 2023. “Comparing Mouse and Human Brains.” eLife 12: e90017. 37428552 10.7554/eLife.90017 PMC10332809

[jnc70160-bib-0200] Xu, N. , T. J. LaGrow , N. Anumba , et al. 2022. “Functional Connectivity of the Brain Across Rodents and Humans.” Frontiers in Neuroscience 16: 816331. 35350561 10.3389/fnins.2022.816331 PMC8957796

[jnc70160-bib-0201] Yang, T. , Y. Luo , W. Ji , and G. Yang . 2021. “Advancing Biological Super‐Resolution Microscopy Through Deep Learning: A Brief Review.” Biophysical Reports 7: 253–266. 10.52601/bpr.2021.210019 PMC10233474 37287757

[jnc70160-bib-0202] Yang, Z. , F. Bertolucci , R. Wolf , and M. Heisenberg . 2013. “Flies Cope With Uncontrollable Stress by Learned Helplessness.” Current Biology 23: 799–803. 23602474 10.1016/j.cub.2013.03.054

[jnc70160-bib-0203] Yemini, E. , A. Lin , A. Nejatbakhsh , et al. 2021. “NeuroPAL: A Multicolor Atlas for Whole‐Brain Neuronal Identification in *C. elegans* .” Cell 184: 272–288.e11. 33378642 10.1016/j.cell.2020.12.012 PMC10494711

[jnc70160-bib-0204] Yi, B. , J. J. Sahn , P. M. Ardestani , et al. 2017. “Small Molecule Modulator of Sigma 2 Receptor Is Neuroprotective and Reduces Cognitive Deficits and Neuroinflammation in Experimental Models of Alzheimer's Disease.” Journal of Neurochemistry 140: 561–575. 27926996 10.1111/jnc.13917 PMC5312682

[jnc70160-bib-0205] Youssef, K. , A. Tandon , and P. Rezai . 2019. “Studying Parkinson's Disease Using *Caenorhabditis elegans* Models in Microfluidic Devices.” Integrative Biology 11: 186–207. 31251339 10.1093/intbio/zyz017

[jnc70160-bib-0206] Zahs, K. R. , and K. H. Ashe . 2010. “‘Too Much Good News’—Are Alzheimer Mouse Models Trying to Tell Us How to Prevent, Not Cure, Alzheimer's Disease?” Trends in Neurosciences 33: 381–389. 20542579 10.1016/j.tins.2010.05.004

[jnc70160-bib-0207] Żakowski, W. 2020. “Animal Use in Neurobiological Research.” Neuroscience 433: 1–10. 32156550 10.1016/j.neuroscience.2020.02.049

[jnc70160-bib-0208] Zhang, Y. , C. Pak , Y. Han , et al. 2013. “Rapid Single‐Step Induction of Functional Neurons From Human Pluripotent Stem Cells.” Neuron 78: 785–798. 23764284 10.1016/j.neuron.2013.05.029 PMC3751803

[jnc70160-bib-0209] Zhang, Y. , S. A. Sloan , L. E. Clarke , et al. 2016. “Purification and Characterization of Progenitor and Mature Human Astrocytes Reveals Transcriptional and Functional Differences With Mouse.” Neuron 89: 37–53. 26687838 10.1016/j.neuron.2015.11.013 PMC4707064

